# Comprehensive Analysis of Secondary Metabolites in *Usnea longissima* (Lichenized Ascomycetes, Parmeliaceae) Using UPLC-ESI-QTOF-MS/MS and Pro-Apoptotic Activity of Barbatic Acid

**DOI:** 10.3390/molecules24122270

**Published:** 2019-06-18

**Authors:** S. Divya Reddy, Bandi Siva, Katragunta Kumar, V. S. Phani Babu, Vemireddy Sravanthi, Joel Boustie, V. Lakshma Nayak, Ashok K Tiwari, CH. V. Rao, B. Sridhar, P. Shashikala, K. Suresh Babu

**Affiliations:** 1 Centre for Natural Products & Traditional Knowledge, CSIR-Indian Institute of Chemical Technology, Tarnaka, Hyderabad 500 007, Telangana, India; divya4474@gmail.com (S.D.R.); bandishiva2008@gmail.com (B.S.); kumarkatragunta@gmail.com (K.K.); tiwari@iict.res.in (A.K.T.); 2 Dept. of Pharmacy, University College of Technology, Osmania University, Hyderabad 500 007, Telangana, India; shashikala_patangay@yahoo.co.in; 3 Centre for NMR & Structural Chemistry, CSIR-Indian Institute of Chemical Technology, Hyderabad 500 007, Telangana, India; vsphanibabu@gmail.com; 4 Organic Synthesis and Process Chemistry Division, CSIR-Indian Institute of Chemical Technology, Hyderabad 500 007, Telangana, India; vemireddy.sravanthireddy@gmail.com; 5 Univ Rennes, CNRS, ISCR (Institut des Sciences Chimiques de Rennes)—UMR 6226, F-35000 Rennes, France; 6 Division of Applied Biology, CSIR-Indian Institute of Chemical Technology, Tarnaka, Hyderabad 500 007, Telangana, India; lakshmanayakiict@gmail.com; 7 Pharmacognosy and Ethnopharmacology Division, CSIR-National Botanical Research Institute Rana Pratap Marg, P.O. Box No. 436, Lucknow 226001, Uttar Pradesh, India; chvrao72@yahoo.com; 8 Analytical Division, CSIR-Indian Institute of Chemical Technology, Tarnaka, Hyderabad 500 007, Telangana, India; bsridhar@csiriict.in

**Keywords:** lichen, *U. longissima*, parmeliaceae, UPLC-ESI-QTOF-MS/MS, cytotoxic activity, cell cycle studies

## Abstract

Considering the importance of ultra-performance liquid chromatography-electrospray ionization-quadrupole time of flight-tandem mass spectrometry (UPLC-ESI-QTOF-MS/MS) hyphenated techniques for analysis of secondary metabolites from crude extracts, the present study was aimed at identification of secondary metabolites in acetone extract of the lichen *Usnea longissima*. From our study, 19 compounds were tentatively identified through comparison of exact molecular masses from their MS/MS spectra, mass fragmentation studies and comparison with literature data. In addition, potent cytotoxic activity of *U. longissima* extract prompted us to isolate four compounds, 18*R*-hydroxy-dihydroalloprotolichesterinic acid (**19**), neuropogolic acid (**20**), barbatic acid (**21**)**,** and usnic acid (**22**) from this extract which were adequately identified through mass spectrometry and NMR spectroscopy. All four compounds displayed cytotoxic activity. Barbatic acid (**21**) manifested doxorubicin equivalent activity against A549 lung cancer cell line with IC_50_ of 1.78 µM and strong G0/G1 accumulation of cells. Poly ADP-ribose polymerase (PARP) cleavage confirmed that it induced cytotoxic activity via apoptosis. Finally, our work has discerned the depside, barbatic acid (**21**) from crude extract as a candidate anti-cancer molecule, which induces cell death by stepping up apoptosis.

## 1. Introduction

Lichens are fungi (mostly Ascomycetes) that feed on populations of microscopic algae/cyanobacteria in the framework of a symbiotic type of relationship. Apart from their ecological importance, they have become important natural medicinal resources due to the production of a large number of unique secondary metabolites (depsides, depsidones, dibenzofurans, pulvinic acid derivatives) and pigments (anthraquinones, napthoquinones, and xanthones) which can act as biomarkers as well as bioactive compounds [1,2,3,4]. *Usnea longissima* Ach. is a hanging hair lichen, that grows circumpolar in high humidity inland areas and coastal forests of Europe, Asia, and North America [5]. This lichen has been used therapeutically for centuries in Indian traditional systems of medicine for its analgesic, cardiotonic, stomachic, and wound healing properties [6]. Its crude extracts are consumed by tribal communities to treat fevers and bone fractures [7]. Previous studies on its chemical constituents have resulted in isolation of several bioactive secondary metabolites which include monosubstituted phenyls, depsides, anthraquinones, dibenzofuran derivatives, and terpenoids [8,9,10]. However, chemical variations are illustrated by the report of seven chemical strains found in the Uttarakhand region, India: (i) barbatic acid and usnic acid (ii) squamatic acid, barbatic acid and usnic acid (iii) diffractaic acid and usnic acid (iv) evernic acid and usnic acid (v) fumarprotocetraric acid and usnic acid (vi) squamatic acid and usnic acid and (vii) usnic acid only [11]. Mallavadhani et al. also reported this species as new source (−)-placodiolic acid along with barbatic acid, ergosterol-5β, 8β-peroxide and (+)-usnic acid from a sample collected in Sikkim, India [12]. Similarly, chemical variations of *U. longissima* are described within the central Coast Range of Oregon [5] as in Southern Far East Russia and in Japan [13]. Most of these depsides and dibenzofuran derivatives (especially usnic acid) are supposed to be responsible for their multiple pharmacological activities [9] and some of its major compounds were absorbed in blood of animals fed with this lichen [14]. 

**Figure 1:**
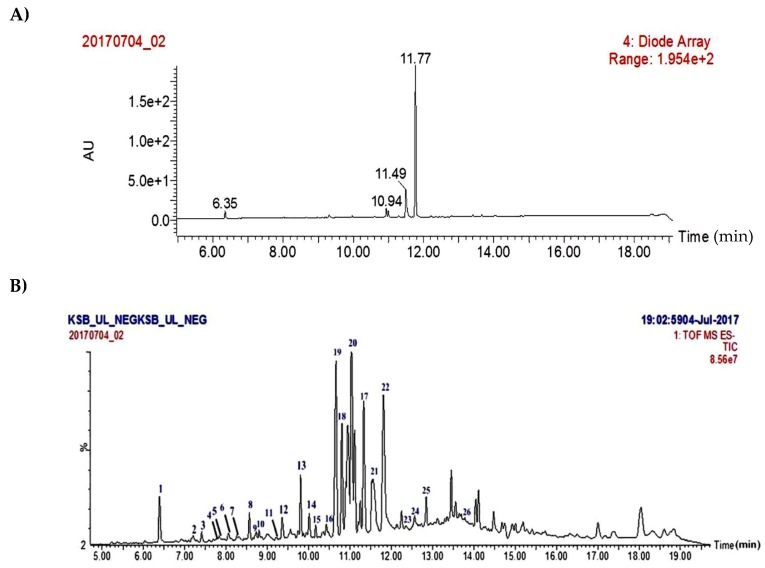
UPLC-PDA (**A**) and TIC Spectra (**B**) (LC-QTOF-MS/MS) of *Usnea longissima* acetone extract.

**Figure 2:**
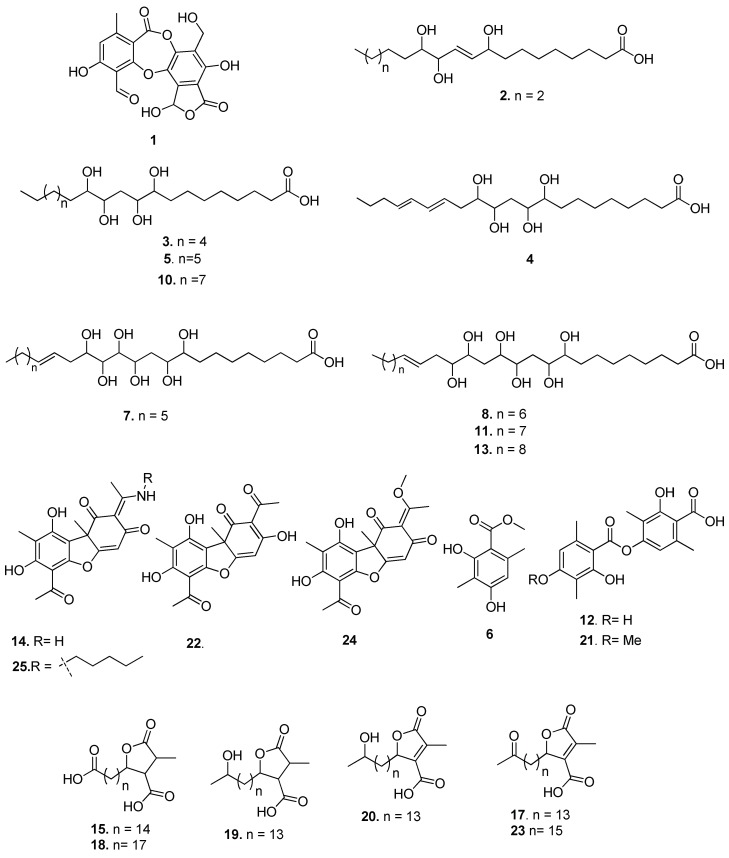
Compounds identified through UPLC-ESI-QTOF-MS/MS in *U. longissima* acetone extract.

**Figure 3:**
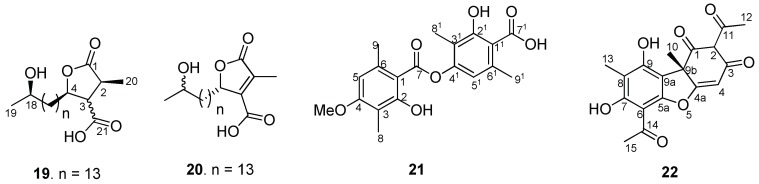
Isolated compounds from *Usnea longissima* acetone extract.

As part of our continuous endeavors of phytochemical–pharmacological integrated studies on Indian lichens for bio-actives [15], a biological screening is developed in parallel to an Ultra Performance Liquid Chromatography-Photo Diode Array-high-resolution Time of Flight Mass Spectrometric (UPLC-PDA-ESI-Q-TOF-MS/MS) method for comprehensive analysis of secondary metabolites in crude acetone extract (Figure 1). Using this analytical technique, 23 secondary metabolites (Figure 2) were characterized from the crude acetone extract through their accurate molecular masses, molecular formula, and comparison of the literature database (DNP) which revealed the presence of unreferenced compounds. Initial screening of the acetone extract of the fruticose lichen *U. longissima* was carried out against cancer cell lines, HeLa (cervical), A549 (lung), MCF-7 (breast), DU-145 (prostate) and HEK-293, a Human embryonic kidney cell line. Interestingly, it was found that the crude extract displayed potent cytotoxic activity, particularly against A-549 cell line. Subsequent bio-guided phytochemical investigation led to the isolation and identification of four compounds (**19**–**22**) (Figure 3). The structures of these compounds (**19**–**22**) were elucidated on the basis of extensive spectroscopic techniques, especially NMR and mass spectrometry data. Single X-ray crystal structure of barbatic acid (**21**) was given for the first time in this study. The aim of our study described here was to identify metabolites responsible for bioactivity of lichen and to explicate their mechanism of action. Therefore, isolates (**19**–**22**) were appraised for cytotoxicity against cancer cell lines. 

## 2. Results and Discussion

### 2.1. Isolation of Major Compounds from Acetone Extract of U. longissima and UPLC/PDA/ESI-QTOF-MS Analysis 

**Figure 4:**
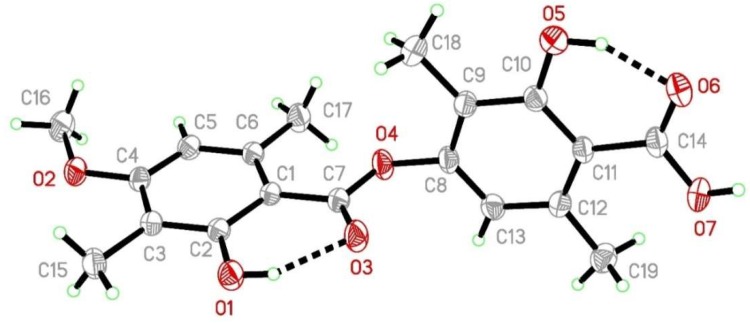
Crystal structure of barbatic acid (**21**): the molecular structure of barbatic acid, with the atom-numbering scheme. Displacement ellipsoids are drawn at the 30% probability level and H atoms are shown as small spheres of arbitrary radius. Intramolecular hydrogen bonds are shown as dashed lines.

Concentrated acetone extract was chromatographed on silica gel and resultant fractions were subjected to bioassay for cytotoxic activity against cancer cell lines. Repeated column chromatography of bioactive fractions resulted in the isolation of four compounds. Comparing their physical and spectroscopic data with literature, they were characterized as 18*R*-hydroxy-dihydroalloprotolichesterinic acid (**19**) [16], neuropogolic acid (**20**) [17], barbatic acid (**21**) [18], and (+)-usnic acid (**22**) (Figure 3) [19]. The structure of barbatic acid (**21**) was further confirmed by single crystal X-ray diffraction analysis (Figure 4). To the best of our knowledge, this is the first report of crystal structure of barbatic acid (**21**).

Recently, Salgado et al. [20] reported finger printing analysis of secondary metabolites from four *Usnea* species (*U. barbata*, *U. antarctica*, *U. rubicunda,* and *U. subfloridana*), using hyphenated techniques. Concerning *U. longissima*, most of the qualitative and quantitative analysis were limited to HPLC or HPTLC studies based on their UV data and retention times. Therefore, in continuation of previous studies on fragmentation patterns on natural products [21] we investigated the crude extract of *U. longissima* via reverse phase UPLC/PDA/ESI-QTOF-MS, using a gradient mobile phase consisting of acetonitrile and water containing 0.1% formic acid that allowed for a comprehensive elution of the analyte within 16 min. Based on these optimized conditions, acquired total ion current chromatograms (TIC) were analyzed in ESI negative ionization mode and these results are presented in Table S1 and Figure 2. Metabolite assignments were made based on their MS spectral data (accurate mass and fragmentation pattern), retention times, comparison to the standard compounds (in-house library), and search in public online-databases (DNP, Reaxys and SciFinder), respectively [22]. This study led to the identification of 23 metabolites distributed in lipids, depsides, depsidones, paraconic acids, and dibenzofuran classes (Figure 2). 

### 2.2. Structural Fragmentation Analysis

All the metabolites were classified based on their common fragmentation ions in MS/MS spectra. Consideration of their UV absorption in PDA and high-resolution masses allowed dereplication of the reference compounds as well as putative characterization of certain secondary metabolites for the first time in the extract. Results are shown in Table S1. Initial TOF-MS/MS scan of the depsidone derivative, salazinic acid (**1**) showed [M − H]^−^ ions at *m/z* 387.0346. Subsequently, ESI-CID-MS/MS analysis of **1** indicated a high abundant product ion [M – H-44]^−^ at *m/z* 343.0455, formed due to loss of CO_2_ which is in line with fragmentation pattern of depsidones [23]. Further, it also showed a fragment ion at *m/z* 313.0346 by loss of CH_2_O, and at *m/z* 269.0451 by loss of CO_2_, respectively confirming the structure of **1** as salazinic acid (Supplementary Figure S5). Similarly, based on MS/MS spectra, compounds **2**, **3**, **4**, **5**, **7**, **8**, **10**, **11**, and **13** were tentatively identified as lipids i.e., polyhydroxy fatty acids, tri-hydroxyfatty acid (**2**), tetra-hydroxy fatty acids (**3**, **4**, **5**, and **10**), and hexa-hydroxy fatty acids (**7**, **8**, **11** and **13**). For explanation of fragmentation pattern, compound **3** was taken as a representative. Its TOF-MS/MS spectra was analyzed, which showed [M − H]^−^ ion at *m/z* 375.2742 (C_20_H_39_O_6_) with key fragment ions 245.1393, 217.1447, 141.0916, and 127.1121 formed by cleavage of C-C bonds (C_8_-C_9_, C_11_-C_12_, C_12_-C_13_) adjacent to hydroxyl groups. The remaining hydroxy fatty acids also followed similar fragmentation pattern along with loss of H_2_O molecules as shown in the Supplementary Material. Further, it was observed that four polyhydroxy fatty acids (**2**, **3**, **5**, **10**) are being reported for the first time in this species.

**Figure 5:**
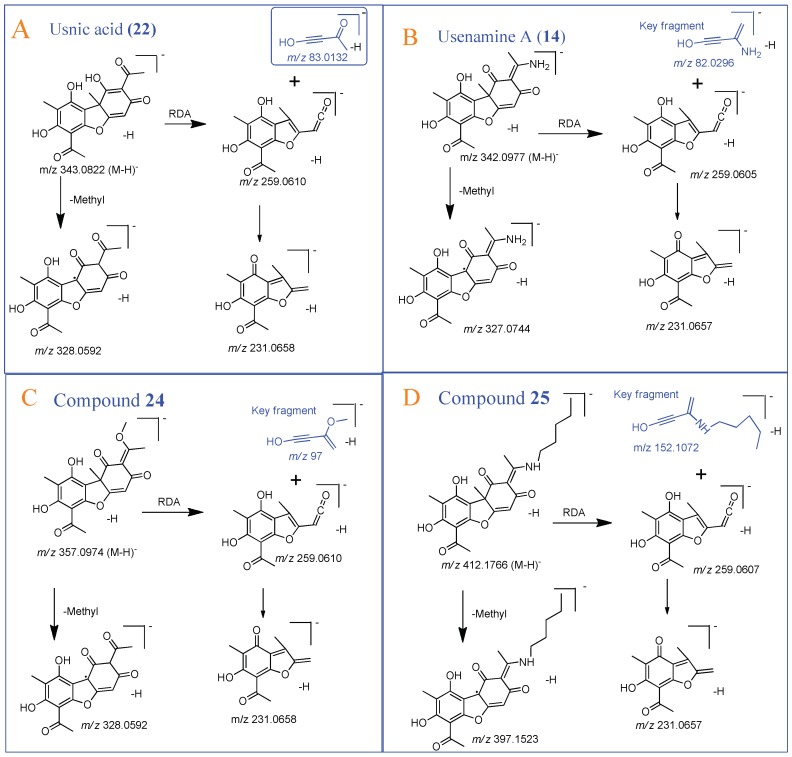
Comparative fragmentation study of (**A**) Usnic acid (**22**), (**B**) Usenamine A (**14**), (**C**) Compound **24** and (**D**) Compound **25**.

ESI-QTOF-MS scan (negative mode) of dibenzofuran related compounds including usnic acid (**22**), usenamine A (**14**) [8], and compounds **24** and **25** showed [M − H]^−^ ion peaks at *m/z* 343.0822 (**22**), 342.0977 (**14**), 357.0974 (**24**), and 412.1766 (**25**) respectively. Usnic acid (**22**) MS/MS spectrum showed a deprotonated ion [M − H]^−^ at *m/z* 343.0822 and an odd electron ion at *m/z* 328, corresponding to [M − H-CH_3_]^−^ [24]. Further, a diagnostic fragment at *m/z* 259 is proposed to be generated from molecular ion *m/z* 343 through Retro Diels Alder (RDA) cleavage of ring C. Fragment at *m/z* 231 was due to the loss of CO from *m/z* 259. Compounds **24** and **25** were similar to that of **22** and **14** respectively, except for the presence of methoxy group in **24** and n-pentyl side chain at NH_2_ in **25**. The characteristic RDA feature of ring C, resulted in product ions at *m/z* 259.0607 and *m/z* 152.1072 with high abundance in MS/MS spectrum of **25**, and fragment ions at *m/z* 97.0284, 259.0604 in MS/MS spectrum of **24**. Thus, based on their MS/MS fragmentation studies (Figure 5), structures of **24** and **25** were tentatively identified. These compounds were not reported in a recent phytochemical study on usnic acid derivatives while additional usenamines, usone, and isousone were isolated and identified from large quantities of *U. longissima* collected in the Sichuan province [8].

Compound **6** is a simple phenolic compound in nature (Supplementary Figure S10) whereas compounds **12** and **21** were depsides, as concluded by fragmentation (Supplementary Figures S16 and S25). Compounds **15**, **17**, **18**, **19**, **20**, and **23** belong to the class of paraconic acid/aliphatic acid-type compounds [20,25] with similar fragmentation pathways and profiles of product ions. Compound **15** was taken for explanation. TOF-MS/MS spectrum indicated the product ions at *m/z* 339.2534, 295.2635, and 253.2164 were formed from molecular ions by sequential CO_2_ loss and aliphatic chain breakage. Loss of hydroxyl-, keto-group and side chains were also observed in some of the compounds.

In the present study, a depsidone, salazinic acid (**1**) which is well reported in *Usnea* genus but rarely in *U. longissima* is a marker of this chemotype [26]. Gagarina et al. have recorded three chemotypes in Yakutia (Russia): (i) medulla with salazinic acid, cortex with usnic acid; (ii) medulla with salazinic, barbatic acid, fatty acids, cortex with usnic acid; (iii) medulla with salazinic, evernic, diffractaic acids, cortex with usnic acid [27]. Based on our HPTLC (Supplementary Figure S3), UPLC-PDA-TOF-MS^e^ (Supplementary Figure S4) and isolation we found usnic acid, barbatic acid, salazinic acid and fatty acids in our sample, which clearly matches with chemotype-2 reported by these authors. 

Among 23 compounds identified by MS/MS, five homologous polyhydroxy fatty acids (**4**, **7**, **8, 11**, **13**), two paraconic acids (**15**, **18**), and two dibenzofuran related metabolites (**24** and **25**) were identified for the first time. Fragmentation pathways (Supplementary Figures S5–S30), molecular formulas, and HR-MS/MS parameters (Supplementary Table S1) are presented in the Supplementary Material and structures are shown in Figure 2.

### 2.3. Biological Activity

Initially, crude acetone extract was screened against a panel of human cancer cell lines, HeLa (cervical), A-549 (lung), MCF-7 (breast), DU-145 (prostate), HEK-293 and a Human embryonic kidney cell line, highlighting a potent inhibitory activity against A549 cell line. Four compounds (**19**–**22**) were isolated from the crude acetone extract of *Usnea longissima* based on a bioassay-guided fractionation and were again tested for their cytotoxicity against those cell lines by MTT assay [28]. The clinically applied anticancer agent, doxorubicin, was used as positive control for cytotoxicity assay. As shown in Table 1, barbatic acid (**21**) manifested potent activity against A549 cell line with IC_50_ value of 1.78 ± 0.62 µM, which is almost equivalent to the standard drug, doxorubicin. However, cytotoxicity value against normal cell line (HEK-293) was found >100 µM. Inhibitory activity of barbatic acid (**21**) on A549 and DU-145 cell lines is comparable to the previously reported activities against MCF-7 (breast cancer) [29] and HeLa (cervix carcinoma) [30] cell lines. This pronounced activity of barbatic acid (**21**) prompted us to investigate its effect on the cell cycle by flow cytometry.

#### 2.3.1. Cell Cycle Analysis

**Figure 6:**
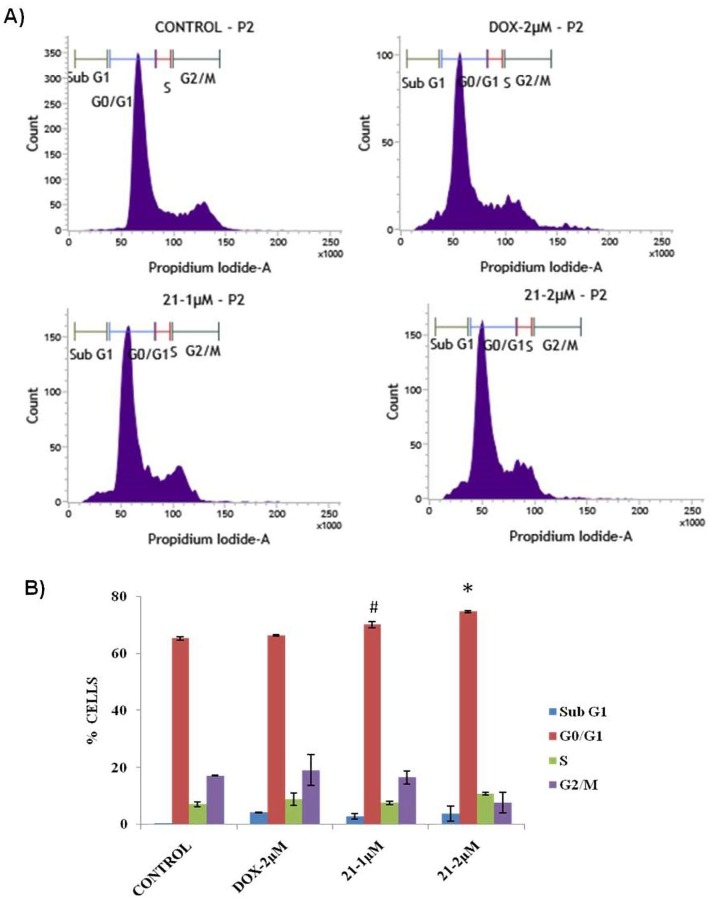
Flow cytometric analysis in A549 cells. (**A**) Control cells and cells treated with Doxorubicin (2 µM) and barbatic acid (**21**) at 1 and 2 µM concentrations. (**B**) Bar chart showing the cell cycle phase distribution of A549 cells. Values are mean ± SE, *n* = 3. Tukey’s multiple comparison test followed by ANOVA was applied to compare the differences. ^#^ *p* < 0.001 (control vs. **21** at 1 µM), * *p* < 0.0001 (control vs. **21** at 2 µM).

The screening results revealed that barbatic acid (**21**) showed significant anticancer activity against human lung cancer cell line, A549. To understand whether this cell growth inhibition of A549 cells was due to cell cycle arrest, we performed a cell cycle analysis study. In this study, A549 cells were treated with barbatic acid (**21**) for 24 h at 1 and 2 µM concentrations. The data obtained clearly indicated that this compound showed cell cycle arrest in G0/G1 phase with 70.9% and 74.4% of cell accumulation in G0/G1 phase at 1 and 2 µM concentration, respectively (Figure 6). 

#### 2.3.2. Hoechst Staining for Apoptosis

**Figure 7:**
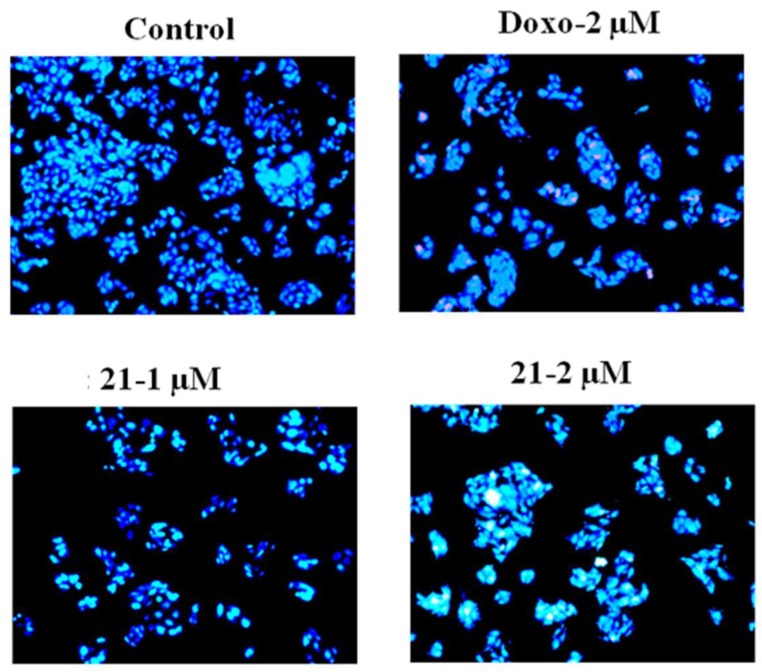
Hoechst staining in the A549 cell line; represents control cells and cells treated with Doxorubicin (2 µM) and barbatic acid (**21**) at 1 and 2 µM concentrations. Florescence images were captured by Olympus microscope using 10X lens.

To validate whether barbatic acid (**21**) indeed caused apoptosis in A549 cells, we analyzed the nuclear fragmentation in treated cells, as one of the characteristics of apoptosis includes chromatin condensation and fragmentation of nuclei in inducing programmed cell death [32]. To achieve this, we treated A549 cells with 1 and 2 µM concentrations of barbatic acid for 48 h. Microscopic examination of Hoechst stained cells revealed chromatin condensation and fragmented nuclei indicating induction of apoptosis in A549 cells due to compound treatment (Figure 7). 

#### 2.3.3. Annexin V Staining

**Figure 8:**
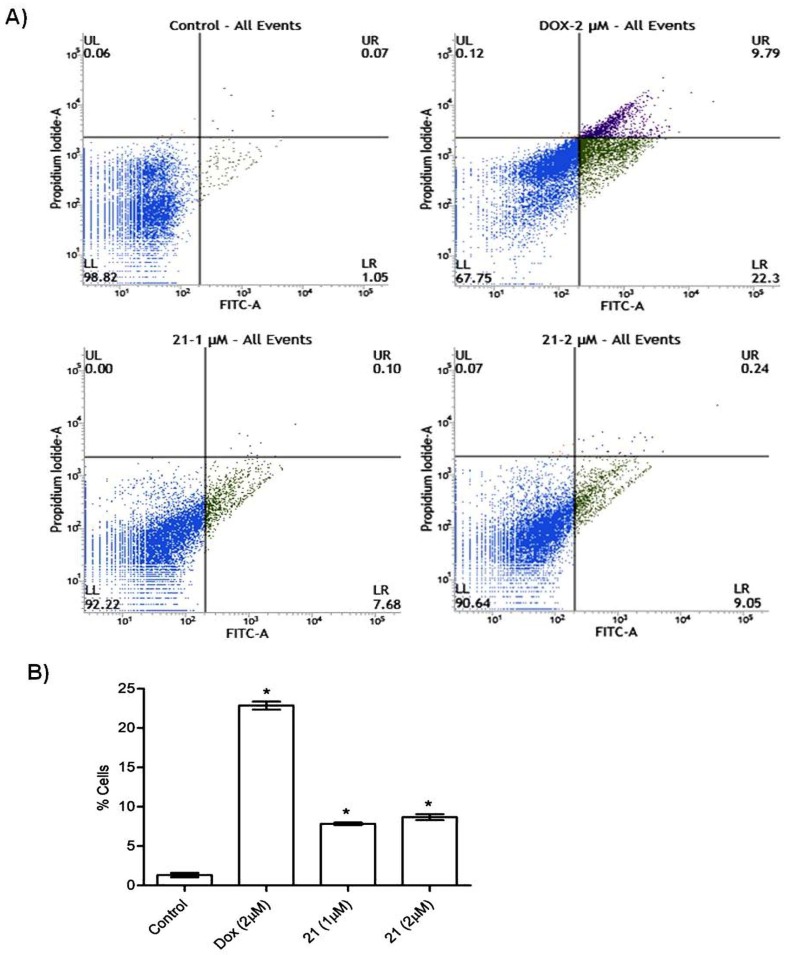
Annexin V-FITC assay in lung cancer cells A549; (**A**) Control cells and cells treated with Doxorubicin (2 µM) and barbatic acid (**21**) at 1 and 2 µM. Lower left (LL) represents live (viable) cells, lower right (LR) represents early apoptotic cells, upper right (UR) represents late apoptotic cells and upper left (UL) represents necrotic cells. (**B**) Bar graph indicating % apoptotic cells. Values are mean ± SD, *n* = 3. Tukey’s multiple comparison test followed by ANOVA was applied to compare the differences. * *p* < 0.0001 vs. control.

Since our flow cytometry and nuclear stain analysis revealed that barbatic acid causes cell death, we corroborated our observation with Annexin V staining. We chose to perform Annexin V staining as, Phosphatidyl serine (PS), a lipid normally confined to the inner leaflet of the plasma membrane, is exported to the outer plasma membrane leaflet during apoptosis to serve as a trigger for recognition of apoptotic cells by phagocytes [33]. Therefore, to elucidate this, A549 cells were treated with barbatic acid (**21**) for 24 h at 1 and 2 µM concentrations. It was observed that compound **21** induced apoptosis by externalizing phosphatidyl serine as evidenced by robust annexin V staining (Figure 8). Furthermore, our results indicated that when compared to control (1.12%), barbatic acid (**21**) showed at about 7.78% and 9.29% total apoptosis at 1 and 2 µM concentrations, respectively (Figure 8).

#### 2.3.4. Immunoblot Assay for Poly (ADP-ribose) polymerase (PARP)

**Figure 9:**
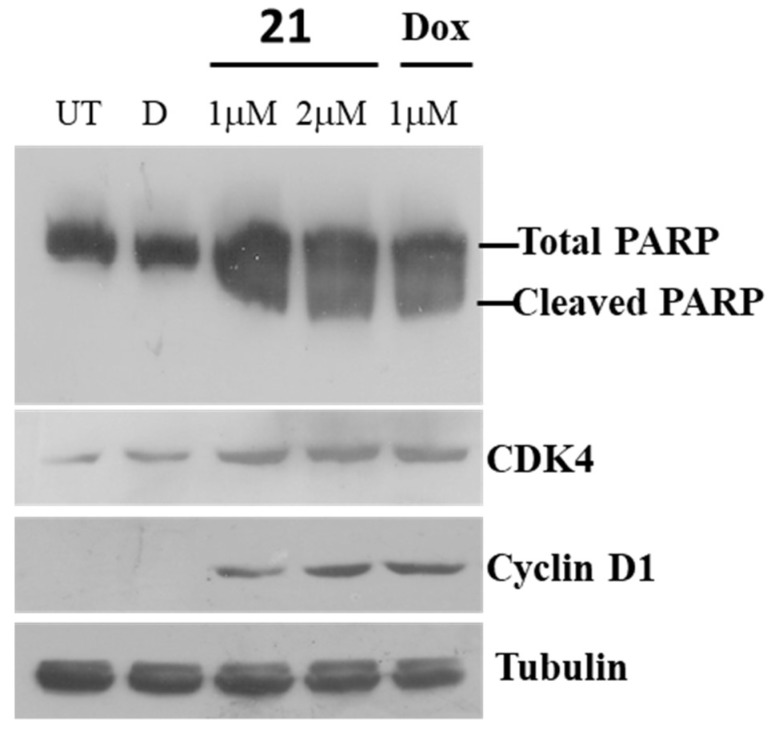
Immunoblot assay of barbatic acid (**21**). A549 cells were seeded and treated with 1 and 2 µM of **21** for 48 h. Subsequently, the cells were harvested, and lysates were subjected to immunoblot analysis. As a positive control, doxorubicin was employed at 1 µM concentration. UT represents untreated cells and D is DMSO, vehicle control. Antibodies were probed against cyclin D1 and CDK4 proteins and tubulin was used as a loading control.

Poly (ADP-ribose) polymerase (PARP) cleavage has been ascribed as one of the hallmarks of apoptosis [34]. Immunoblot analysis revealed that **21** led to robust cleavage of PARP protein similar to doxorubicin treatments (Figure 9). In addition, we analyzed for CDK4 and Cyclin D1 protein levels, as they promote the transition of cell cycle from G1 to S. CDK4 and Cyclin D1 protein levels accumulated in compound **21** and doxorubicin treated cells (Figure 9). 

#### 2.3.5. Caspase-3 Assay

**Figure 10:**
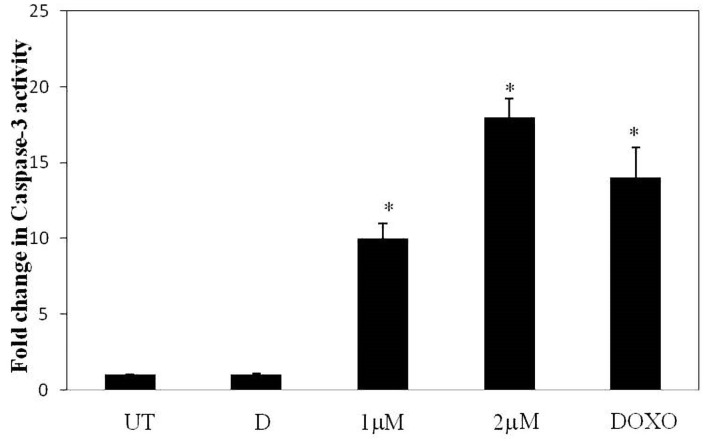
Caspase-3 assay of barbatic acid (**21**). A549 cells were seeded and treated with 1 and 2 µM of **21**. Subsequently, the cells were harvested, and lysates were subjected to cellular caspase 3 analysis. As a positive control doxorubicin was employed. UT represents untreated cells and D is DMSO, vehicle control. Values are mean ± SD, the assay was repeated two times in triplicates (*n* = 6). * *p* < 0.05 vs. control is treated as statistically significant.

To further confirm that compound **21** indeed causes cell death, we sought to determine intracellular activities of caspase-3. To achieve this, we treated A549 cells with 1 and 2 µM concentrations of **21** with doxorubicin as a positive control and performed fluorimetric assays. As expected, caspase-3 activity increased maximally in 2 µM of **21** treated cells. Whereas, untreated or vehicle treated cells did not show any activation in caspase-3 levels (Figure 10). 

Thus, we could show that barbatic acid (**21)** is a potent cytotoxic agent which causes apoptosis through accumulation of cells in G0/G1, activation of caspase-3 that correlates with appearance of cleaved PARP protein.

## 3. Materials and Methods

### 3.1. Lichen Sample

*U. longissima* (Common name: Old Man’s beard) was collected in the month of November 2013 from Osla region between altitude range 3000–3500 and Hari-ki-dun region between altitude range 3500–4000 m in Uttarkashi district of Uttarakhand state of India [11,35]. Specimens were recorded with accession no. 28,382 (lichen from Osla), and no. 12-018850 (lichen from Hari-ki-dun) and deposited in the CSIR-National Botanical Research Institute (Lucknow, India) Herbarium. The lichen was authenticated by Prof. D. K. Upreti, and a dissecting microscope was used for identification of morphological characteristics of the thallus, reproductive structures, color, size and shape. Spot test reactions were performed on the thallus under the dissecting microscopy. Spot test by *para-*phenylenediamine (P), potassium hydroxide (K), sodium hypochlorite (C) or/and KC reagent characters and comparison to the standard literature [27,36] were referred for identification of lichen samples (Supplementary Figure S2). Three major lichen substances were visualized with high performance thin layer chromatography in toluene/Dioxan/Acetic acid—72.50:24.19:3.22 solvent system (Supplementary Figure S3).

### 3.2. Chemicals and Reagents 

HPLC-grade acetonitrile was obtained from Biosolve Chimie SARL (Dieuze, France), formic acid (Optima LC/MS grade) from Fisher Scientific (Geel, Belgium), and methanol (LiChrosolv) was purchased from Merck (Darmstadt, Germany). Ultra-pure water (Milli-Q, Millipore, Milford, MA, USA) was used throughout the study. 

### 3.3. Extraction and Purification of Constituents from U. longissima

The lichen *U. longissima* (300 g) was shade dried, powdered and extracted with acetone at room temperature for 48 h. Resulting acetone extract was evaporated to dryness under reduced pressure affording syrupy residue (13 g). Crude extract was subjected to gradient column chromatography (silica gel, 100–200 mesh, eluting with hexane/EtOAc mixtures of increasing polarity) to give four major fractions (F1–F4). Repeated purifications of fraction F1 on silica gel (100–200 mesh) with elution of hexane:EtOAc (60:40 *v/v*) yielded (+) usnic acid (**22**) (1.5 g) [8,19] in pure form. Similarly, purification of fraction F2 was achieved by flash chromatography with elution of hexane:EtOAc (50:50 *v/v*) followed by recrystallization to yield barbatic acid (**21**) (30 mg) [18]. Repetitive purification of fraction F3 on silica gel (100–200 mesh and 230–400 mesh) on elution with hexane:EtOAc (50:50 *v/v*) yielded 18*R*-hydroxy-dihydroalloprotolichesterinic acid (**19**) (25 mg) [16]. Fraction F4 was then purified on silica gel (100–200 mesh) and elution with hexane:EtOAc (50:50 *v/v*) yielded neuropogolic acid (**20**) (5 mg) [17].

### 3.4. Spectral Data

*18R-hydroxy-dihydroalloprotolichesterinic acid* (**19**): White solid; [α]_D_^25^ −8.00 (c = 0.1, CHCl_3_); ^1^H-NMR (700 MHz in Acetone D_6_) δ 2.95 (1H, m, H-2), 3.29 (1H, dd, *J* = 8.9, 8.3, H-3), 4.70 (1H, ddd, *J* = 8.0, 8.5, 5.8, H-4), 1.28 (2H, m, H-6), 1.28 to 1.24 (18H, m, H-7 to 15), 1.28 (2H, m, H-16), 1.59 (2H, m, H-17), 3.67 (1H, m, H-18), 1.10 (3H, d, *J* = 6.1, H-19), 1.22 (3H, d, *J* = 7.1, H-20); ^13^C-NMR (175 MHz in Acetone D_6_) δ 177.1 (C-1), 36.4 (C-2), 51.1 (C-3), 77.2 (C- 4), 30.9 (C-5), 25.65 (C-6), 29.0- 28.0 (C-7 to 15), 25.6 (C-16), 39.4 (C-17), 66.6 (C-18), 23.1 (C-19), 13.7 (C- 20), 174.3 (C-21); ESI-MS *m/z* 369 [M − H]^−^.

*Neuropogolic acid* (**20**): White solid; [α]_D_^25^ +20.6 (c = 0.09, CHCl_3_); ^1^H-NMR (700 MHz in CDCl_3_ + CD_3_OD) δ 5.15 (1H, m, H-4), 1.45 to 1.62 (22H, m, H-7 to 17), 3.72(1H, m, H-18), 1.15 (3H, d, *J* = 6.1, H-19), 2.16 (3H, d, *J* = 2.0, H-20); ^13^C-NMR (75 MHz in CDCl_3_ + CD_3_OD) δ 172.3 (C-1), 139.0 (C-2), 146.6 (C-3), 81.0 (C-4), 32.2 (C-5), 24.2 (C-6), 29.1- 28.8 (C-7 to 15), 23.4 (C-16), 43.4 (C-17), 66.5 (C-18), 29.1 (C-19), 10.5 (C-20), 165.1(C-21) ; ESI-MS *m/z* 391 [M + Na]^+^.

*Barbatic acid* (**21**): White crystal; ^1^H NMR (500 MHz in DMSO-D_6_) δ 10.73 (1H, s, 2′-OH), 3.86 (3H, s, 4- OMe), 6.59 (IH, s, H-5), 6.68 (1H, s, H-5′), 1.99 (3H, s, H-8), 2.00 (3H, s, H-8′), 2.48(3H, s, H-9), 2.56 (3H, s, H-9′). ^13^C-NMR (100 MHz in DMSO-D_6_) δ 106.2 (C-1), 161.0 (C-2), 109.9 (C-3), 161.2 (C-4), 55.6 (4-OCH_3_), 106.9 (C-5), 138.9 (C-6), 168.5 (C-7), 9.0 (C-8), 23.0 (C-9), 115.6 (C-1′), 159.4 (C-2′), 111.34 (C-3′), 151.7 (C-4′), 115.9 (C-5′), 138.9 (C-6′), 173.0 (C-7′), 7.9 (C-8′), 22.7 (C-9′).; ESI-MS *m/z* 359 [M − H]^−^.

*Usnic acid* (**22**): Yellow solid; [α]_D_^25^ + 163.9 (c = 0.05, CHCl_3_); ^1^H-NMR (500 MHz in CDCl_3_): δ 5.98 (H-4), 13.31 (1H, s, 7-OH), 11.03 (1H, s, 9-OH), 1.76 (3H, s, H-10), 2.66 (3H, s, H-12), 2.11 (3H, s, H-13), 2.68 (3H, s, H-15); ^13^C-NMR (75 MHz in CDCl_3_) δ 198.0 (C-1), 105.1 (C-2), 191.69 (C-3), 98.27 (C-4), 179.29 (C-4a), 101.44 (C-6), 155.15 (C- 6a), 169.8 (C-7),109.3 (C-8), 157.42 (C-9), 103.88 (C-9a), 59.0 (C-9b), 32.0 (C-10), 201.7(C-11), 27.87 (C-12), 7.5 (C-13), 200.29 (C-14), 31.24 (C-15); ESI-MS *m/z* 343 [M − H]^−^.

### 3.5. Instrumental UPLC Conditions

High-resolution masses of secondary metabolites were measured after UPLC separation. Chromatographic separation was performed on Acquity H Class UPLC system (Waters, Milford, MA, USA) with a conditioned auto sampler, using an Acquity BEH C_18_ column (100 × 2.1 mm id., 1.7 μm particle size) (Waters, Milford, MA, USA). Column temperature was maintained at 40 °C. Mobile phase consisting of water with 0.1% formic acid in water (solvent A) and acetonitrile with 0.1% formic acid (solvent B) was pumped at a flow rate of 0.4 mL/min. Gradient elution program was as follows: 0 min, 5% B; 3.00 min, 20% B; 5.00 min, 35% B; 7.50 min, 50% B; 10.00 min, 70% B; 11.50 min, 85% B; 12.50 min 95% B; 17.00 min 95% B; 18.00 min 5% B. Equilibration time was 2.8 min and injection volume was 1 μL. LC-QTOF-MS^e^ mode was applied to analyze the samples in both TIC as well as MS/MS mode where collision energy was ramped at 15–45 eV. Eluted compounds were detected from *m/z* 50 to 1200 using Xevo G2-XS QTOF mass spectrometer (Waters, Manchester, UK), which was connected to Electrospray ionization (ESI) interface with negative ion mode using the following instrument settings, capillary voltage, 2.0 KV; sample cone, 40 V; source temperature, 120 °C; desolvation temperature 350 °C; cone gas flow rate 50 L/h; desolvation gas (N_2_) flow rate 850 L/h, Argon as CID gas for MS/MS experiments. All analyses were performed using the lock spray, which ensured accuracy and reproducibility. Leucine-Enkephalin (5 ng/mL) was used as lock mass, generating a reference ion in negative mode at *m/z* 554.2615 introduced by a lock spray at 10 μL/min for accurate mass acquisition. Data acquisition was achieved using Mass lynx v 4.1 (Waters, Milford, MA, USA). Acquiring data in this manner provided information of intact precursor ions as well as fragment ions.

### 3.6. Biological Activity

#### 3.6.1. Cytotoxic Activity 

MTT assay was performed as described earlier [28,37]. Cells were plated at a density of 1 × 10^4^ cells/well in 100 µL Dulbecco’s Modified Eagle Medium (DMEM)/Minimum Essential Media (MEM) medium, supplemented with 10% FBS in 96-well plates for 24 h. After incubation, cells were treated with standard doxorubicin and test compounds for 48 h. After 48 h of treatment, 10 µL MTT (3-(4,5-dimethylthiazol-2 yl)-2,5-diphenyl tetrazolium bromide) (5 mg/mL) were added to each well and the plates were further incubated for 4 h. After 4 h, 100 µL DMSO was added to each well and absorbance at 570 nm wavelength was recorded spectrophotometrically. 

#### 3.6.2. Cell Cycle Analysis 

Flow cytometric analysis (FACS) was performed to evaluate the cell cycle arrest. A549 cells were seeded, incubated for 24 h and then treated with doxorubicin at 2 µM and compound **21** at 1 and 2 µM concentrations for 24 h. After that, cells were harvested and washed with phosphate buffered saline (PBS) followed by fixation in ice cold 70% ethanol. After fixation cells were incubated with RNase A (0.1 mg/mL) at 37 °C for 30 min and stained with propidium iodide (Sigma Aldrich, St. Louis, MO, USA) (50 μg/mL) for 30 min on ice in dark. Cell cycle analysis was performed by flow cytometry (Becton Dickinson FACS Caliber instrument) [32]. 

#### 3.6.3. Hoechst Staining

A549 cells were seeded on cover slips and incubated for 24 h. After incubation, cells were treated with doxorubicin at 2 µM and compound **21** at 1 and 2 µM concentrations for 48 h. After 48 h treatment, Hoechst staining was performed using H33258 dye [38]. Florescence images were captured by Olympus microscope using 10X lens.

#### 3.6.4. Annexin V Assay for Apoptosis 

A549 cells were seeded in six-well plates and incubated for 24 h. After incubation, cells were treated with doxorubicin at 2 µM and compound **21** at 1 and 2 µM concentrations and further incubated for 24 h. After 24 h treatment, cells were harvested by trypsinization. After harvesting, cells were washed with PBS at 5000 rpm. After washing, cells were stained with Annexin-V and propidium iodide using Annexin-V apoptosis detection kit (Sigma-Aldrich). Annexin V-FITC assay was performed as described earlier [39]. 

#### 3.6.5. Western Blotting Assay

Following treatments, cells were washed twice with PBS and lysed in 1× SDS sample buffer. Proteins were separated on 10% SDS-polyacrylamide gels and transferred onto nitrocellulose membranes. Membranes were washed twice with tween Tris-buffered saline before blocking nonspecific binding with 5% nonfat dry milk (Blotto, Santa Cruz Biotechnology, Dallas, TX, USA) or 3% bovine serum albumin. Anti-Poly ADP-ribose Polymerase (PARP) (11835238001-Roche) antibody, that detects total and cleaved PARP was used at 1:1000 dilutions, and membranes were incubated for 2 h at room temperature. Anti-Cyclin D1 (C7464, Sigma Aldrich) and anti-CDK4 (SAB140559, Sigma Aldrich) were detected similar to PARP protein. Membranes were washed three times, and detection was performed using horseradish peroxidase-conjugated secondary antibody as described previously [40]. 

#### 3.6.6. Caspase-3 Assay

Following treatment, cell lysates were washed twice with phosphate-buffered saline and dissolved in lysis buffer (50 mM HEPES (pH 7.4) containing 5 mM CHAPS and 5 mM DTT) for 15 min on ice. Later, cells were centrifuged at 14,000× *g* for 15 min, under cold conditions, and collected the clear supernatant. The supernatant was added to assay buffer (20 mM HEPES (pH 7.4), 2 mM EDTA, 0.1% CHAPS, and 5 mM DTT) containing caspase 3 substrate (Ac-DEVD-AMC, 40 μM) in a final volume of 100 μL as described earlier [41]. The incubation was performed for 1 h at 37 °C with readings recorded at 5-min intervals. Fluorescence released by AMC was measured at 360-nm and 460-nm excitation and emission wavelengths, respectively. Values were normalized to protein concentration and expressed as fold change of activity relative to DMSO control [40].

#### 3.6.7. Statistical Analysis

For cell cycle analysis and Annexin assays, one-way ANOVA followed by Tukey’s multiple comparison tests was applied to compare the differences within the groups. Statistical analyses were performed using GraphPad Prism version 5.01 (GraphPad Software Inc., San Diego, CA, USA). For caspase activity, statistical differences were calculated using Student’s *t* test and *p* values for pair wise comparisons were calculated by employing a two-tailed *t* test. *p* < 0.05 was considered significant for all the assays.

### 3.7. X-ray Crystal Data of ***21***

X-ray data was collected at room temperature using a Bruker Smart Apex CCD diffractometer with graphite monochromated MoKα radiation (λ = 0.71073Å) with ω-scan method [42]. Preliminary lattice parameters and orientation matrices were obtained from four sets of frames. Integration and scaling of intensity data were accomplished using the SAINT program. Structures were solved by direct methods using SHELXS97 and refinement was carried out by full-matrix least-squares technique using SHELXL97 [43]. Anisotropic displacement parameters were included for all non-hydrogen atoms. The O bound H atoms were located in difference Fourier maps and their positions and isotropic displacement parameters were refined. All other H atoms were positioned geometrically and treated as riding on their parent C atoms, with C-H distances of 0.93–0.96 Å, and with U_iso_(H) = 1.2U_eq_ (C) or 1.5U_eq_ for methyl atoms [42]. 

Crystal Data for C_19_H_20_O_7_ (*M* = 360.37 g/mol): triclinic, space group P-1 (no. 2), *a* = 7.7896(16) Å, *b* = 7.8083(16) Å, *c* = 14.169(3) Å, *α* = 90.052(3)°, *β* = 99.293(3)°, *γ* = 92.053(3)°, *V* = 849.9(3) Å^3^, *Z* = 2, *T* = 294.15 K, μ(Mo Kα) = 0.108 mm^−1^, *Dcalc* = 1.4080 g/cm^3^, 9792 reflections were measured (2.92° ≤ 2Θ ≤ 56.82°), 3961 unique (*R*_int_ = 0.0207, R_sigma_ = 0.0205), which were used in all calculations. The final *R*_1_ was 0.0539 (I ≥ 2u(I)) and *wR*_2_ was 0.1890 (all data). CCDC 1,831,752 contains supplementary crystallographic data for the structure. These data can be obtained free of charge at www.ccdc.cam.ac.uk/conts/retrieving.html (or from the Cambridge Crystallographic Data Centre (CCDC), 12 Union Road, Cambridge CB2 1EZ, UK; fax: +44(0) 1223 336 033; email: deposit@ccdc.cam.ac.uk) [43].

## 4. Conclusion

As a support to studies reporting cytotoxicity of barbatic acid on cancer cell lines (HEp-2, NCI-H292 and KB) [44], our study confirmed that barbatic acid (**21**) has a marked activity (IC_50_ 1.78 µM) against the A549 cancer cell line and moderate activity (IC_50_ = 3.02–9.03 µM) on additional cancer cell lines (HeLa, MCF-7 and DU-145) with some selectivity (IC_50_ = 124.9µM on HEK-293 embryonic cell line). Various lines of evidence from our study support that barbatic acid (**21**) is a pro-apoptotic agent with arrest in G0/G1 phase. Amongst them, annexin V and Hoechst staining demonstrated that barbatic acid (**21**) causes cell death through nuclear damage and induces apoptosis by robust cleavage of PARP protein. Further, a comprehensive analysis of acetone extract from *U. longissima* was carried out using hyphenated UPLC-MS/MS for the first time which resulted in the identification and characterization of 23 metabolites based on their accurate molecular masses and molecular formula. Among them, compounds **1** and **6** were identified by MS/MS by comparison with standards and compounds **19**–**22** were identified by MS/MS by comparison with isolated compounds. Nine compounds including five lipids, two paraconic acids, and two usnic acid derivatives were putatively identified for the first time by MS/MS, and fragmentation patterns supporting their structures were proposed. Such a chemical study is mandatory when chemotypes are concerned and could explain some differences in efficacy or uses of traditional herbal medicines. In this case, barbatic acid is abundant in the *U. longissima* chemotype studied but this common lichen species can have great variations in its chemical profile. Finally, our findings strongly suggest that barbatic acid (**21**) is a real cytotoxic agent against A549 cells and can be considered as a new drug candidate to be assessed on oncogene-specific aggressive lung cancers.

## Figures and Tables

**Table 1:** IC_50_ values for acetone extract and isolated compounds **19**–**22** on selected human cell lines.

	HeLa ^a^	A549 ^b^	MCF-7 ^c^	DU-145 ^d^	HEK-293 ^e^
Crude extract	25.33 ± 1.23	9.11 ± 2.76	14.67 ± 2.85	20.88 ± 3.74	78.33 ± 2.11
**19**	22.67 ± 0.97	20.20 ± 1.99	20.01 ± 0.74	26.97 ± 2.92	55.87 ± 1.55
**20**	23.63 ± 0.74	19.13 ± 3.86	22.30 ± 4.05	26.10 ± 1.09	112.77 ± 3.82
**21**	3.02 ± 0.11	1.78 ± 0.62	3.16 ± 0.88	9.03 ± 2.11	124.90 ± 2.65
**22**	18.88 ± 2.06	13.80 ± 1.11	19.18 ± 2.05	24.21 ± 0.99	125.20 ± 1.98
Doxorubicin	1.55 ± 1.32	1.95 ± 0.97	1.62 ± 0.36	1.51 ± 0.84	NT

50% cell proliferation inhibitory concentration (IC_50_) after 48 h of compound treatment. IC_50_ values presented for extract is in µg/mL and for compounds in µM. **^a^** Human cervical cancer, **^b^** Human lung cancer, **^c^** Human breast cancer, **^d^** Human prostate cancer and **^e^** Normal cell lines. NT: Not tested [31].

## Supplementary Materials

Supplementary materials are available online.
