# Supplementary material for: Comprehensive Analysis of Secondary Metabolites in Usnea longissima (Lichenized Ascomycetes, Parmeliaceae) Using UPLC-ESI-QTOF-MS/MS and Pro-Apoptotic Activity of Barbatic Acid
Source: Molecules. 2019 Jun 18;24(12):2270. doi: 10.3390/molecules24122270 (PMC6630668; doi:10.3390/molecules24122270)
Supplement: Supplementary file 1 [file molecules-24-02270-s001.pdf]

Supplementary materials

Comprehensive Analysis of Secondary Metabolites in *Usnea longissima* (Lichenized Ascomycetes, Parmeliaceae) Using UPLC-ESI-QTOF-MS/MS and Pro-Apoptotic Activity of Barbatic Acid

S. Divya Reddy [1,2], Bandi Siva [1], Katragunta Kumar [1], V. S. Phani Babu [3], Vemireddy Sravanthi [4], Joel Boustie [5,*], V. Lakshma Nayak [6], Ashok K Tiwari [1], CH. V. Rao [7], B. Sridhar [8], P. Shashikala [2] and K. Suresh Babu [1,*]

[1] Centre for Natural Products & Traditional Knowledge, CSIR-Indian Institute of Chemical Technology, Tarnaka, Hyderabad 500 007, Telangana, India

[2] Dept. of Pharmacy, University College of Technology, Osmania University, Hyderabad-500 007,Telangana, India

[3] Centre for NMR & Structural Chemistry, CSIR-Indian Institute of Chemical Technology, Hyderabad 500 007, Telangana, India

[4] Organic Synthesis and Process Chemistry Division, CSIR-Indian Institute of Chemical Technology, Hyderabad 500 007, Telangana, India

[5] Univ Rennes, CNRS, ISCR (Institut des Sciences Chimiques de Rennes) - UMR 6226, F-35000 Rennes, France

[6] Division of Applied Biology, CSIR-Indian Institute of Chemical Technology, Tarnaka, Hyderabad-500 007, Telangana, India

[7] Pharmacognosy and Ethnopharmacology Division, CSIR-National Botanical Research Institute Rana Pratap Marg, P.O. Box No. 436, Lucknow 226001, Uttar Pradesh, India

[8] Analytical Division, CSIR-Indian Institute of Chemical Technology, Tarnaka, Hyderabad-500 007, Telangana, India

* Correspondence: joel.boustie@univ-rennes1.fr (J.B.); suresh@iict.res.in (K.S.B.); Tel: +91-40-27191881 (K.S.B.); Fax: +91-40-27160512 (K.S.B.)

Identification of *U. longissima*:

Microscopic study showed *Usnea longissima* is pale greenish or silvery-yellowish-green, fruticose and pendulous; main branches are cylindric, up to 3 meters or more in length and very rarely dividing, with numerous dense, short perpendicular side branches and fibrils of about equal length (3-40 mm). The cortex is smooth but disintegrating on the main stems, leaving rough patches of white medulla over the pinkish-to-brownish central cord. The unexposed central cord beneath the cortex is white (when exposed with a razor blade), but frequently turns pinkish or reddish brown in decorticate main branches.

Central cord I+B. Cortex and medulla K-, C-, KC- PD- (with various combinations of evernic, diffractaic, barbatic, and 4-O-demethylbarbatic acids; sometimes with usnic acid only), rarely K+Y-O, PD+O (salazinic acid).

Usnea longissima is distinguished from other *Usnea* species by the extremely long, mostly unbranched main strands, which have perpendicular side branches and fibrils, and a patchy surface due to the eroded cortex. Small specimens may be confused with other pendant *Usnea* species, but only *U. longissima* has a I+ violet or dark bluish central cord and eroding cortex.

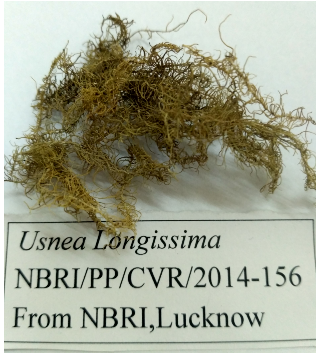

Figure S1. *Usnea longissima* lichen specimen.

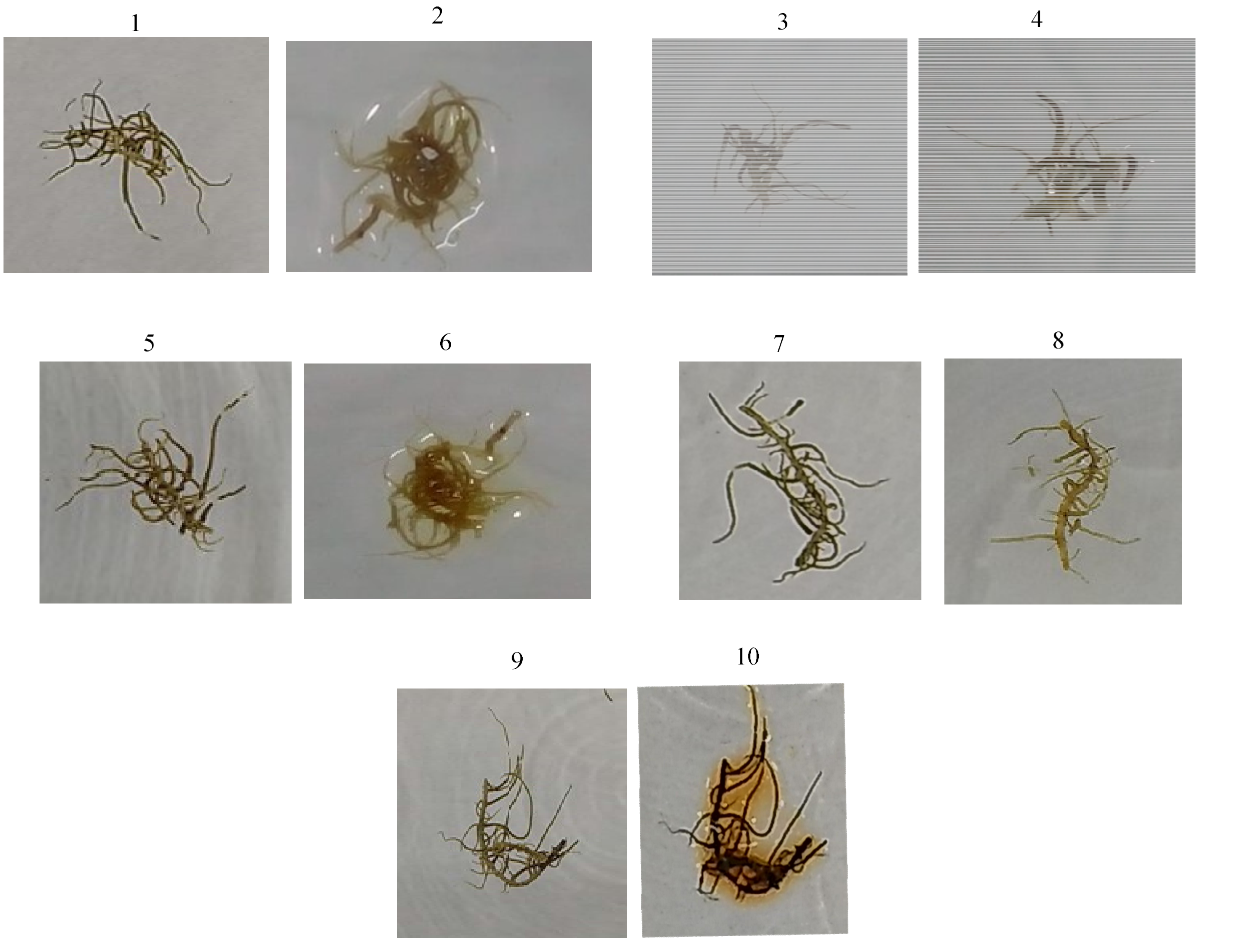

Figure S2. *U. longissima* spot tests for identification (1, 3, 5, 7, 9 = authentic *Usnea longissima*; 2 = after K test, 4 = after C test, 6 = after KC test, 8= after CK test and 10 = after Pd test)

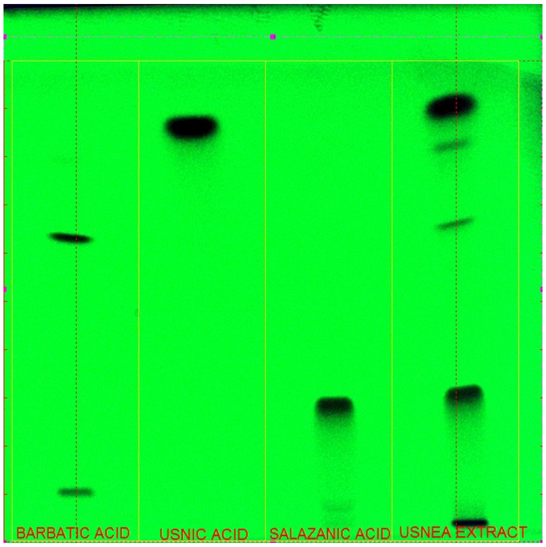

Figure S3. HPTLC Profiles for *U. longissima extract* along the standard compounds at 254 nm, TLC system: Toluene / Dioxan / Acetic acid - 72.50: 24.19 : 3.22

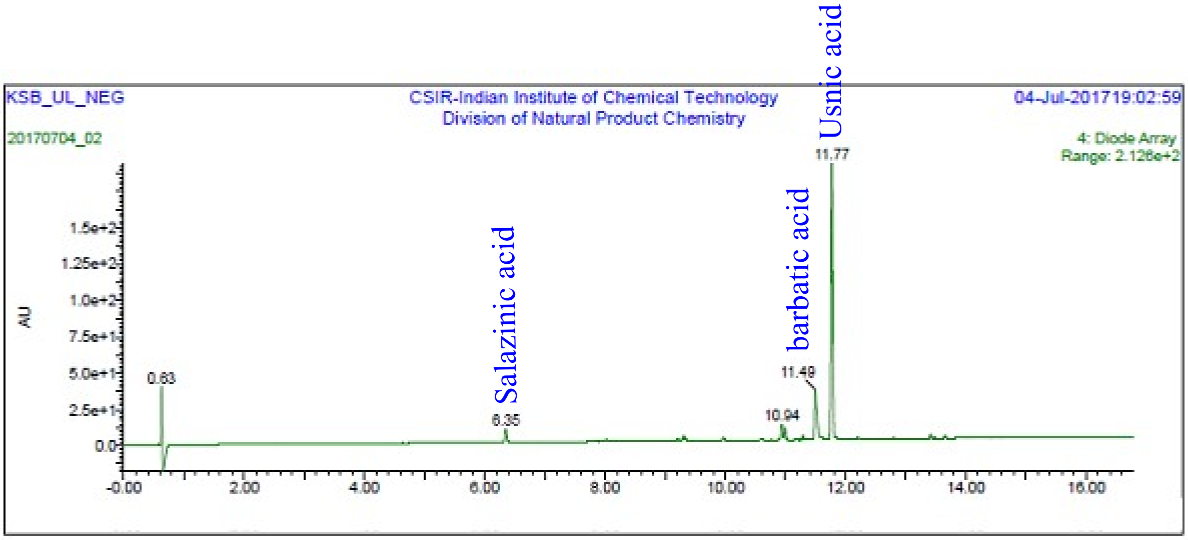

Figure S4. UPLC-PDA chromatogram of acetone extract of *U. longissima*

Table S1. LC-ESI-QTof-MS^e (-ve mode) data of compounds from acetone extract of *Usnea longissima* and their product ions

**Compound Number**	**Compound**	**Ret time (min)**	**Monoisotopic mass [M-H]$^-$ *m/z***	**Molecular ion [M-H]$^-$ Formula (Error) (ppm)**	**Fragment ions (Molecular formula) [M-H]$^-$**
1	[#] Salazinic acid	6.39	387.0346	$C_{18}H_{11}O_{10}$ (-1.6)	343.0453($C_{17}H_{11}O_8$), 325.0346($C_{17}H_9O_7$), 313.0346($C_{16}H_9O_7$), 299.0552($C_{16}H_{11}O_6$), 269.0540($C_{15}H_9O_5$), 253.0497($C_{15}H_9O_4$), 243.0294($C_{13}H_7O_5$), 227.0344($C_{13}H_7O_4$), 177.0183($C_9H_5O_4$), 165.0184($C_8H_5O_4$), 151.0390($C_8H_7O_3$), 121.0285($C_7H_5O_2$)
2	Trihydroxy-octadecenoic acid	7.20	329.2324	$C_{18}H_{33}O_5$ (-1.2)	311.2222 ($C_{18}H_{31}O_4$), 293.2113 ($C_{18}H_{29}O_3$), 229.1439 ($C_{12}H_{21}O_4$), 211.1331 ($C_{12}H_{19}O_3$), 193.1230 ($C_{12}H_{17}O_2$), 83.1380 ($C_{11}H_{19}O_2$), 171.1018 ($C_9H_{15}O_3$), 139.1118 ($C_9H_{15}O$), 127.1118 ($C_8H_{15}O$), 99.0808 ($C_6H_{11}O$)
3	Tetrahydroxy-eicosanoic acid	7.41	375.2742	$C_{20}H_{39}O_6$	357.2639 ($C_{20}H_{37}O_5$), 339.2536 ($C_{20}H_{35}O_4$), 321.2431($C_{20}H_{33}O_3$), 289.1816 ($C_{18}H_{25}O_3$) 275.1499 ($C_{13}H_{23}O_6$), 245.1393 ($C_{12}H_{21}O_5$), 217.1447 ($C_{11}H_{21}O_4$), 199.1334 ($C_{11}H_{19}O_3$) 187.0968 ($C_9H_{15}O_4$), 181.1216 ($C_{11}H_{17}O_2$), 157.0865 ($C_8H_{13}O_3$), 141.0916 ($C_8H_{13}O_2$), 127.1121 ($C_8H_{15}O$), 113.0996 ($C_7H_{13}O$)
4	[o] Unreported compound	7.82	385.1481	$C_{18}H_{25}O_9$ (-4.7)	299.1111 ($C_{14}H_{19}O_7$), 281.1005 ($C_{14}H_{17}O_6$) 213.0737 ($C_{10}H_{13}O_5$), 195.0632 ($C_{10}H_{11}O_4$)
5	Tetrahydroxy-heneicosanoic acid	7.85	389.2890	$C_{21}H_{41}O_6$ (-3.3)	371.2785 ($C_{21}H_{39}O_5$), 353.2667 ($C_{21}H_{37}O_4$), 335.2575 ($C_{21}H_{35}O_3$), 259.1543 ($C_{13}H_{23}O_5$), 231.1598 ($C_{12}H_{23}O_4$), 213.1483 ($C_{12}H_{21}O_3$), 201.1119 ($C_{10}H_{17}O_4$), 195.1379 ($C_{12}H_{19}O_2$), 171.1019 ($C_9H_{15}O_3$), 157.1221 ($C_9H_{17}O_2$), 127.1101 ($C_8H_{15}O$)

6	[#] Methyl β-orsellinate	8.09	195.0655	$C_{10}H_{11}O_4$ (-1.0)	180.0419 ($C_9H_8O_4$), 151.0754 ($C_9H_{11}O_2$), 136.0521 ($C_8H_8O_2$)
7	º Unreported compound	8.30	475.3263	$C_{25}H_{47}O_8$ (-1.7)	461.3107 ($C_{24}H_{45}O_8$), 415.3051 ($C_{23}H_{43}O_6$), 403.3055 ($C_{22}H_{43}O_6$), 385.2968 ($C_{22}H_{41}O_5$), 243.1589 ($C_{13}H_{23}O_4$), 225.1488 ($C_{13}H_{21}O_3$), 215.1284 ($C_{11}H_{19}O_4$), 185.1591 ($C_{12}H_{21}O$), 157.1240 ($C_9H_{17}O_2$)
8	º Unreported compound	8.56	489.3411	$C_{26}H_{49}O_8$ (-3.3)	447.3313 ($C_{24}H_{47}O_7$), 429.3206 ($C_{24}H_{45}O_6$), 411.3105 ($C_{24}H_{43}O_5$), 393.2995 ($C_{24}H_{41}O_4$), 383.3136 ($C_{23}H_{43}O_4$), 347.2935 ($C_{23}H_{39}O_2$), 331.3004 ($C_{23}H_{39}O$), 271.1919 ($C_{15}H_{27}O_4$), 253.1792 ($C_{15}H_{25}O_3$), 241.1431 ($C_{13}H_{21}O_4$), 197.1534 ($C_{12}H_{21}O_2$), 167.1422 ($C_{11}H_{19}O$), 59.0131 ($C_2H_3O_2$)
9	Unidentified	8.69	375.1074	$C_{19}H_{19}O_8$ (-1.6)	359.0766 ($C_{18}H_{15}O_8$), 341.0657 ($C_{18}H_{13}O_7$), 326.0426 ($C_{17}H_{10}O_7$), 311.0192 ($C_{16}H_7O_7$), 257.0445 ($C_{14}H_9O_5$), 181.0501 ($C_9H_9O_4$), 163.0394 ($C_9H_7O_3$)
10	Tetrahydroxy tricosanoic acid	8.80	417.3212	$C_{23}H_{45}O_6$ (-0.7)	403.3049 ($C_{22}H_{43}O_6$), 399.3104 ($C_{23}H_{43}O_5$), 381.3003 ($C_{23}H_{41}O_4$), 259.1906 ($C_{14}H_{27}O_4$), 241.1804 ($C_{14}H_{25}O_3$), 229.1440 ($C_{12}H_{21}O_4$), 201.1177 ($C_{12}H_{17}O_3$), 199.1336 ($C_{11}H_{19}O_3$), 185.1176 ($C_{10}H_{17}O_3$), 183.1395 ($C_{11}H_{19}O_2$), 155.1433 ($C_{10}H_{19}O$), 127.1128 ($C_{18}H_{15}O$), 99.0817 ($C_6H_{11}O$)
11	º Unreported compound	9.19	503.3630	$C_{27}H_{51}O_8$ (9.1)	461.3467 ($C_{25}H_{49}O_7$), 443.3365 ($C_{25}H_{47}O_6$), 425.3244 ($C_{25}H_{45}O_5$), 241.1442 ($C_{13}H_{21}O_4$), 171.1381 ($C_{10}H_{19}O_2$)
12	4-O-Demethylbarbatic acid	9.35	345.0974	$C_{18}H_{17}O_7$ (0.0)	181.0501 ($C_9H_9O_4$), 163.0392 ($C_9H_7O_3$), 137.0600 ($C_8H_9O_2$)
13	º Unreported compound	9.81	517.3734	$C_{28}H_{53}O_8$ (-1.2)	475.3626 ($C_{26}H_{51}O_7$), 457.3530 ($C_{26}H_{49}O_6$), 439.3418 ($C_{26}H_{47}O_5$), 421.3325 ($C_{26}H_{45}O_4$), 411.3470 ($C_{25}H_{47}O_4$), 403.3204 ($C_{26}H_{43}O_3$),

					359.3311 ($C_{25}H_{43}O$), 329.1956 ($C_{17}H_{29}O_6$), 271.1916 ($C_{15}H_{27}O_4$), 241.1439 ($C_{13}H_{21}O_4$), 197.1541 ($C_{12}H_{21}O_2$), 185.1537 ($C_{11}H_{21}O_2$), 167.1434 ($C_{11}H_{19}O$), 155.1426 ($C_{10}H_{19}O$), 59.0134 ($C_2H_3O_2$)
14	Usenamine A	10.01	342.0977	$C_{18}H_{16}NO_6$ (-0.3)	327.0744 ($C_{17}H_{13}NO_6$), 312.0513 ($C_{16}H_{10}NO_6$), 301.0712 ($C_{16}H_{13}O_6$), 286.0477 ($C_{15}H_{10}O_6$), 259.0605 ($C_{14}H_{11}O_5$), 231.0657 ($C_{13}H_{11}O_4$) 139.0388 ($C_7H_7O_3$), 82.0296 (C_4H_4NO)
15	º Unreported compound	10.19	383.2430	$C_{21}H_{35}O_6$ (-1.0)	339.2534 ($C_{20}H_{35}O_4$), 315.2525 ($C_{18}H_{35}O_4$), 295.2635 ($C_{19}H_{35}O_2$), 279.2318 ($C_{18}H_{31}O_2$) 253.2164 ($C_{16}H_{29}O_2$), 181.1589 ($C_{12}H_{21}O$)
16	Unidentified	10.42	315.2530	$C_{18}H_{35}O_4$ (-1.6)	297.2424 ($C_{18}H_{33}O_3$), 279.2321 ($C_{18}H_{31}O_2$), 267.2313 ($C_{17}H_{31}O_2$), 251.2365 ($C_{17}H_{31}O$), 239.2371 ($C_{16}H_{31}O$), 201.1122 ($C_{10}H_{17}O_4$), 171.1019 ($C_9H_{15}O_3$), 155.1069 ($C_9H_{15}O_2$), 141.1274 ($C_9H_{17}O$), 127.1119 ($C_8H_{15}O$)
17	Isomuronic acid	11.3	365.2338	$C_{21}H_{33}O_5$ (2.7)	321.2445 ($C_{20}H_{33}O_3$), 277.2549 ($C_{19}H_{33}O$), 261.2224 ($C_{18}H_{29}O$), 233.2266 ($C_{17}H_{29}$), 223.2064 ($C_{15}H_{27}O$), 207.2114 ($C_{15}H_{27}$), 183.1749 ($C_{12}H_{23}O$), 169.1591 ($C_{11}H_{21}O$), 155.1434 ($C_{10}H_{19}O$), 123.0811 ($C_8H_{11}O$)
18	º Unreported compound	10.80	425.2900	$C_{24}H_{41}O_6$ (-0.7)	413.2901 ($C_{23}H_{41}O_6$), 399.2747 ($C_{22}H_{39}O_6$), 355.2870 ($C_{21}H_{39}O_4$), 337.3107 ($C_{22}H_{41}O_2$), 311.2960 ($C_{20}H_{39}O_2$), 269.2131 ($C_{16}H_{29}O_3$), 201.1268 ($C_{14}H_{17}O$), 195.1396 ($C_{12}H_{19}O_2$) 157.0856 ($C_8H_{13}O_3$)
19	* 18*R* hydroxy dihydroalloprotolichesterinic acid	10.67	369.2641	$C_{21}H_{37}O_5$ (0.0)	325.2746 ($C_{20}H_{37}O_3$), 279.2692 ($C_{19}H_{35}O$), 265.2534 ($C_{18}H_{33}O$), 113.0236 ($C_5H_5O_3$)
20	* Neuropogolic acid	11.05	367.2489	$C_{21}H_{35}O_5$ (1.4)	323.2588 ($C_{20}H_{35}O_3$), 295.2636 ($C_{19}H_{35}O_2$), 279.2689 ($C_{19}H_{35}O$), 277.2533 ($C_{19}H_{33}O$), 263.2375 ($C_{18}H_{31}O$), 235.2421 ($C_{17}H_{31}$),

					207.2110 ($C_{15}H_{27}$), 169.1588 ($C_{11}H_{21}O$), 155.1431 ($C_{10}H_{19}O$), 113.0239 ($C_5H_5O_3$)
21	* Barbatic acid	11.63	359.1134	$C_{19}H_{19}O_7$ (0.8)	181.0504 ($C_9H_9O_4$), 163.0394 ($C_9H_7O_3$), 137.0600 ($C_8H_9O_2$), 119.0492 (C_8H_7O)
22	* Usnic acid	11.88	343.0822	$C_{18}H_{15}O_7$ (1.2)	328.0592 ($C_{17}H_{12}O_7$), 313.0352 ($C_{16}H_9O_7$), 299.0920 ($C_{17}H_{15}O_5$), 259.0610 ($C_{14}H_{11}O_5$), 231.0658 ($C_{13}H_{11}O_4$), 139.0395 ($C_7H_7O_3$) 83.0132 ($C_4H_3O_2$)
23	Vinapraesorediosic acid A	12.40	393.2633	$C_{23}H_{37}O_5$ (2.0)	349.2740 ($C_{22}H_{37}O_3$), 305.2838 ($C_{21}H_{37}O$), 279.2699 ($C_{19}H_{35}O$), 235.2420 ($C_{17}H_{31}$), 211.2081 ($C_{14}H_{27}O$), 197.1905 ($C_{12}H_{25}O$)
24	º Unreported compound	12.59	357.0974	$C_{19}H_{17}O_7$ (0.0)	342.0737 ($C_{18}H_{14}O_7$), 314.0425 ($C_{16}H_{10}O_7$), 301.0710 ($C_{16}H_{13}O_6$), 286.0481 ($C_{15}H_{10}O_6$), 273.0759 ($C_{15}H_{13}O_5$), 259.0604 ($C_{14}H_{11}O_4$) 245.0812 ($C_{14}H_{13}O_4$), 231.0653 ($C_{13}H_{11}O_4$) 189.0546 ($C_{11}H_9O_3$), 97.0284 ($C_5H_5O_2$), 83.0136 ($C_4H_3O_2$)
25	º Unreported compound	12.96	412.1766	$C_{23}H_{26}NO_6$ (1.5)	397.1523 ($C_{22}H_{23}NO_6$), 380.1498 ($C_{22}H_{22}NO_5$), 368.1137 ($C_{20}H_{18}NO_6$), 354.0977 ($C_{19}H_{16}NO_6$), 341.0906 ($C_{18}H_{15}NO_6$), 326.0667 ($C_{17}H_{12}NO_6$), 312.0510 ($C_{16}H_{10}NO_6$), 259.0606 ($C_{14}H_{11}O_5$) 231.0657 ($C_{13}H_{11}O_4$), 152.1072 ($C_9H_{14}NO$)
26	Unidentified	13.87	631.4926	$C_{39}H_{67}O_6$ (-1.9)	587.5027 ($C_{38}H_{67}O_4$), 543.5126 ($C_{37}H_{67}O_2$), 369.2632 ($C_{21}H_{37}O_5$), 351.2533 ($C_{21}H_{35}O_4$), 325.2738 ($C_{20}H_{37}O_3$), 307.2634 ($C_{20}H_{35}O_2$), 279.2320 ($C_{18}H_{31}O_2$), 261.2215 ($C_{18}H_{29}O$) 113.0234 ($C_5H_5O_3$)

*Compared with isolated compounds.

# Compared with standard compounds.

º Tentatively identified.

Table S2: Structures of the compounds tentatively identified by UPLC-ESI-QTof-MS[e]

	Compound name	Structure
1	Salazinic acid	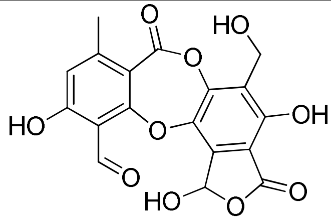
2	Trihydroxy-octadecenoic acid	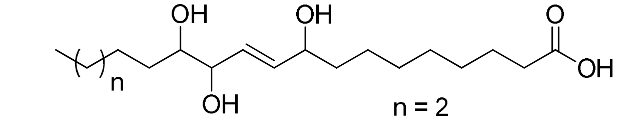
3	Tetrahydroxy-eicosanoic acid	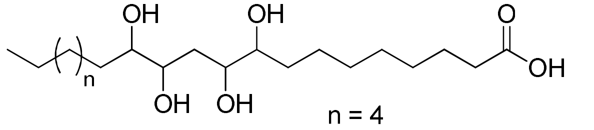
4	Unreported compound	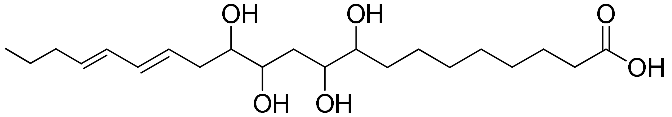
5	Tetrahydroxy-heneicosanoic acid	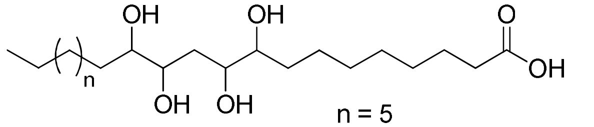
6	Methyl β-orsellinate	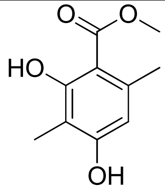
7	Unreported compound	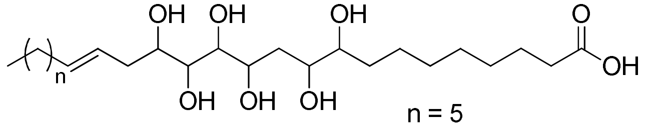
8	Unreported compound	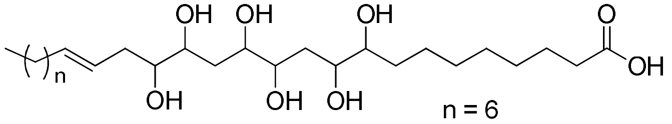
10	Tetrahydroxy tricosanoic acid	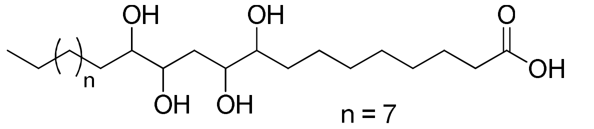
11	Unreported compound	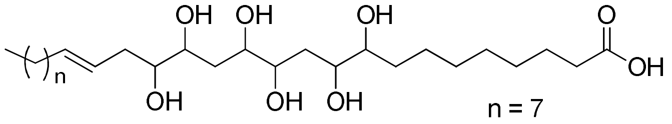

12	4-O-Demethylbarbatic acid	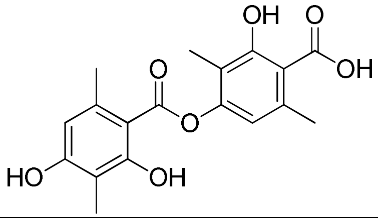
13	Unreported compound	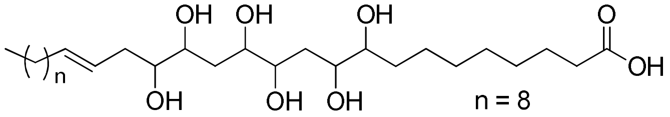
14	Usenamine A	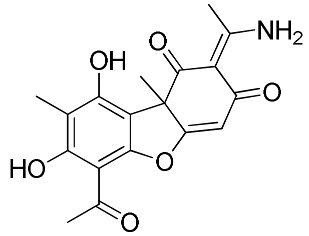
15	Unreported compound	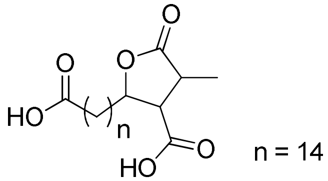
17	Isomuronic acid	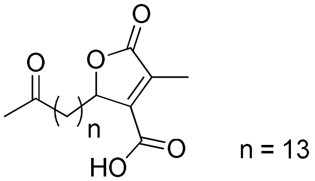
18	Unreported compound	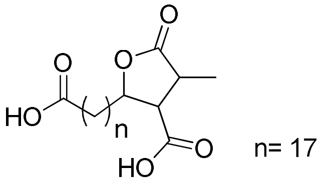
19	18*R* hydroxy dihydroalloprotolichesterinic acid	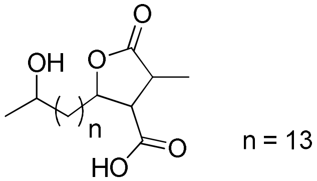
20	Neuropogolic acid	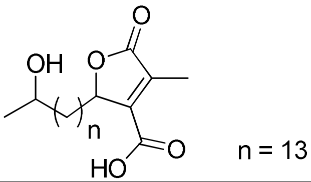
21	Barbatic acid	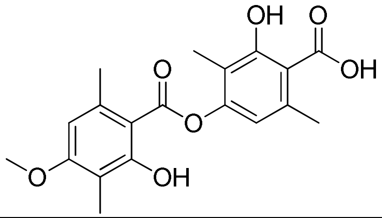

22	Usnic acid	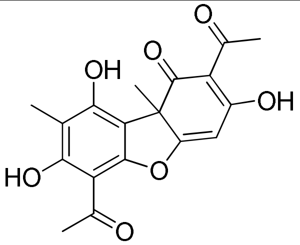
23	Vinapraesorediosic acid A	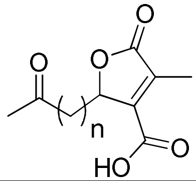
24	Unreported compound	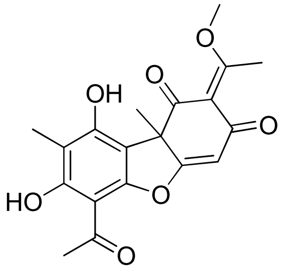
25	Unreported compound	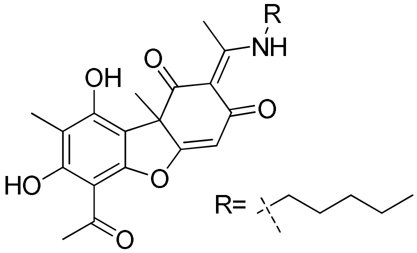

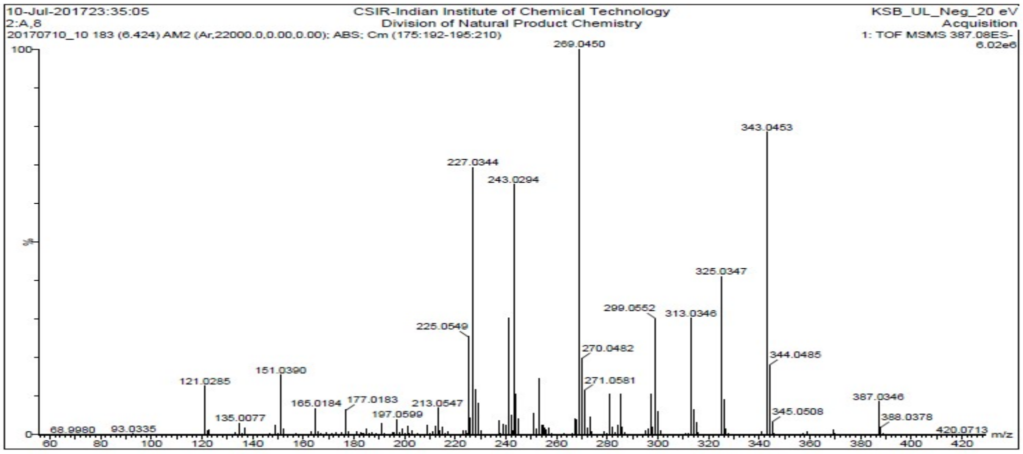

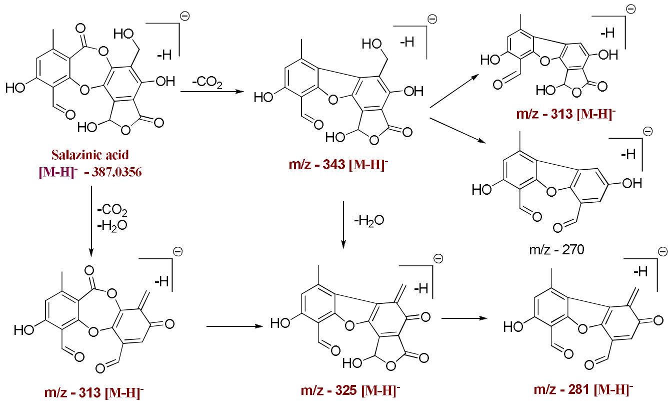

Figure S5. MS/MS spectrum and proposed fragmentation of compound **1**

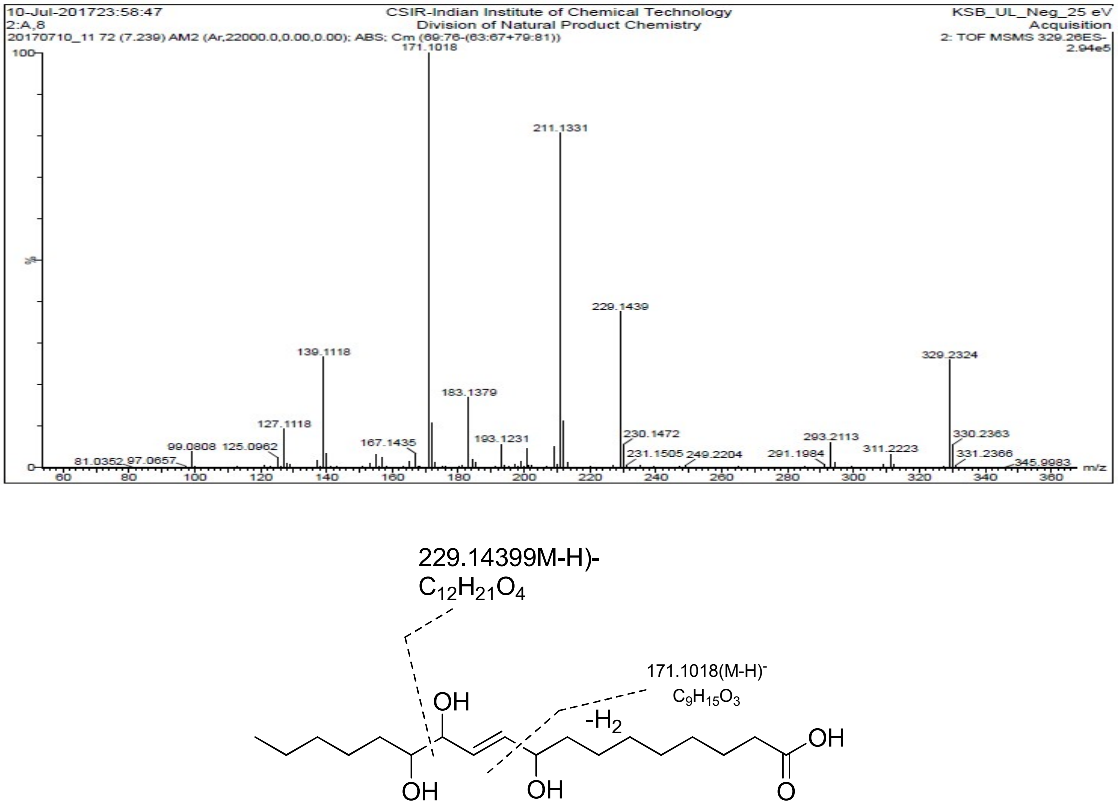

Figure S6. MS/MS spectrum and proposed fragmentation of compound **2**

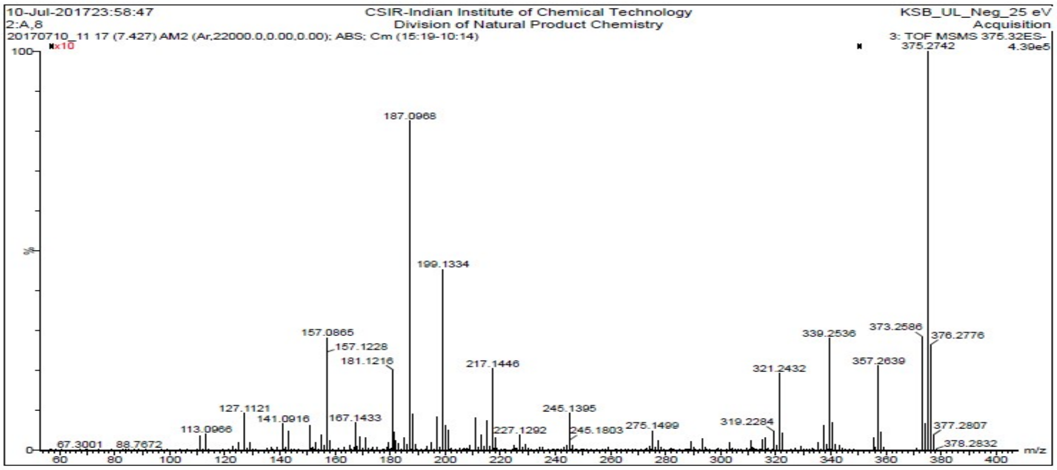

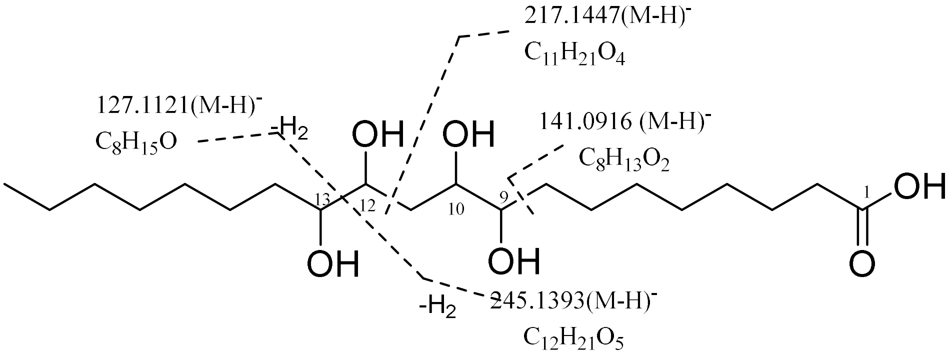

Figure S7. MS/MS spectrum and proposed fragmentation of compound **3**

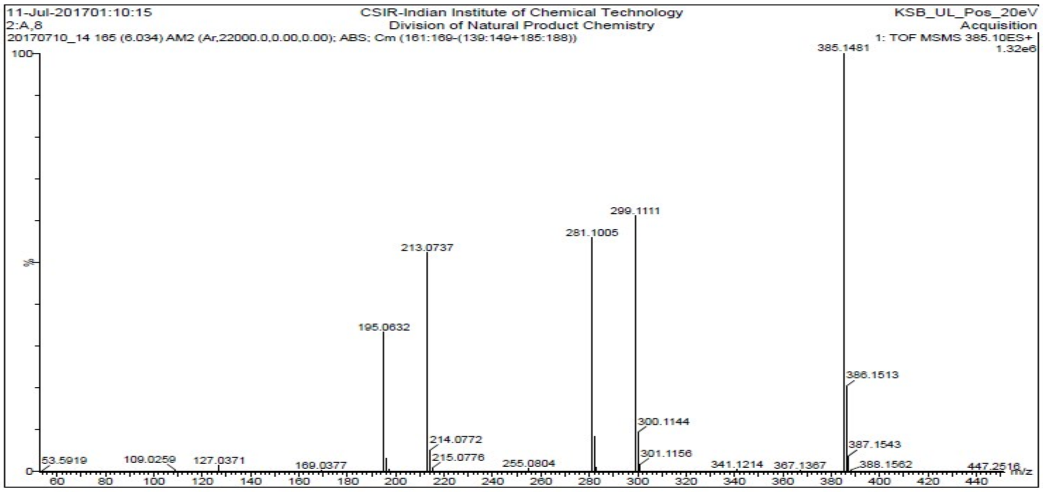

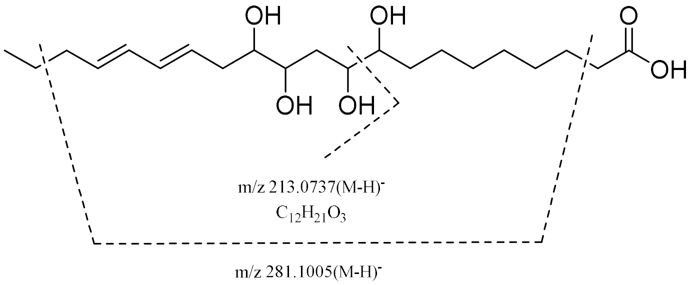

Figure S8. MS/MS spectrum and proposed fragmentation of compound **4**

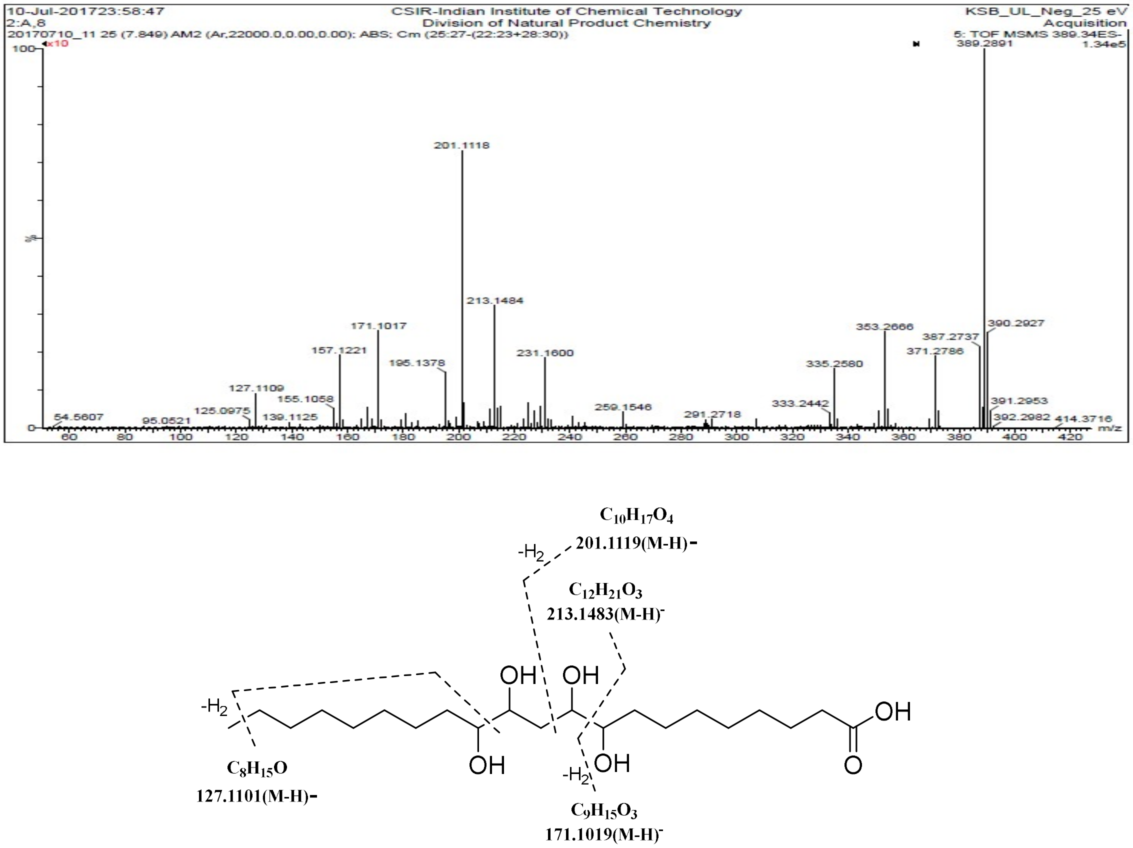

Figure S9. MS/MS spectrum and proposed fragmentation of compound **5**

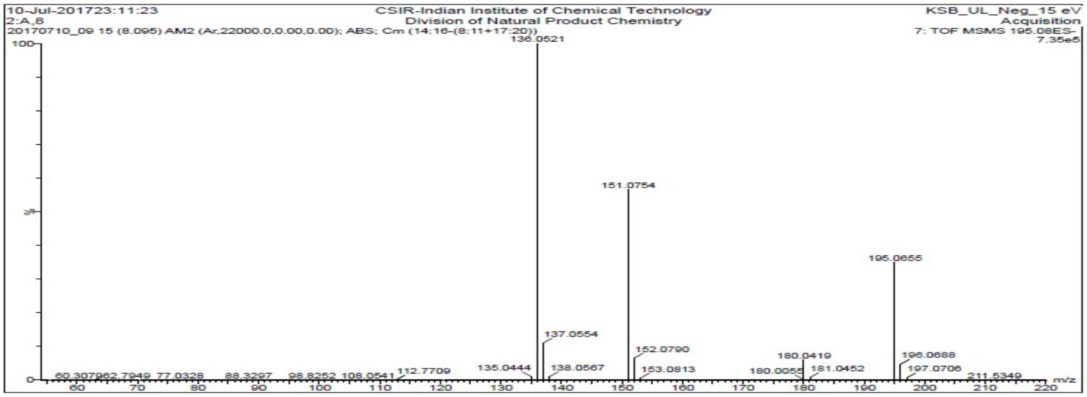

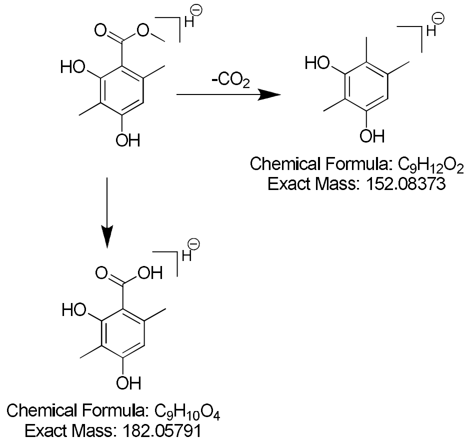

Figure S10. MS/MS spectrum and proposed fragmentation of compound **6**

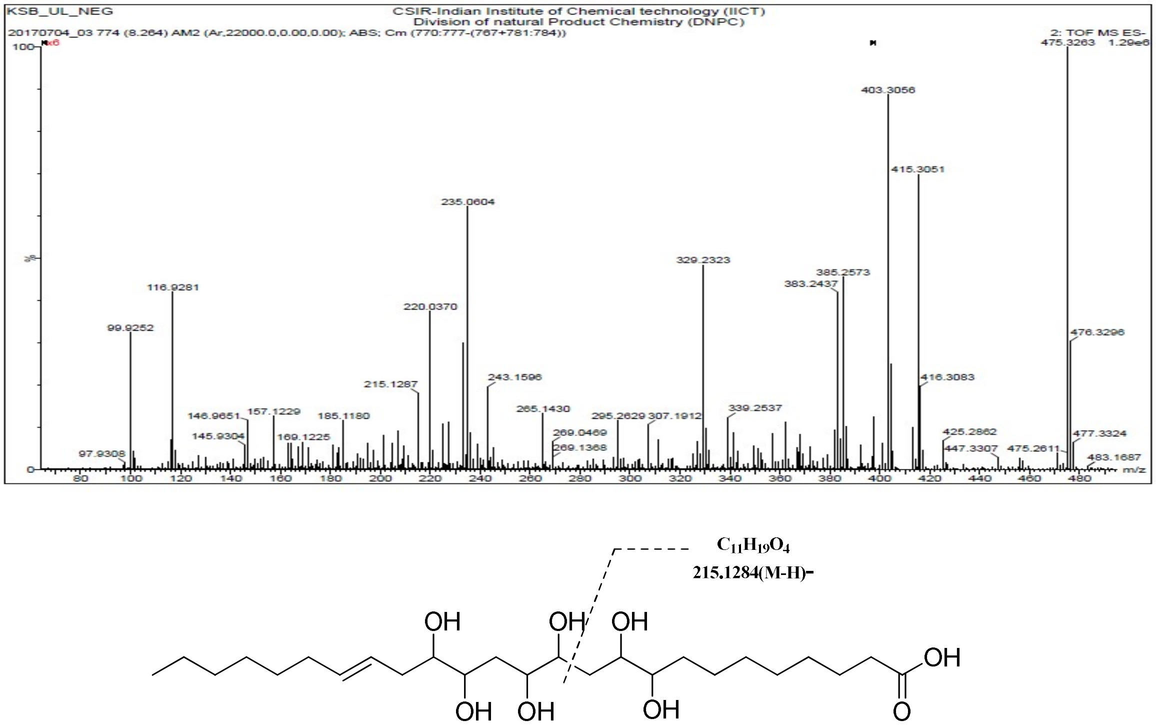

Figure S11. MS/MS spectrum and proposed fragmentation of compound **7**

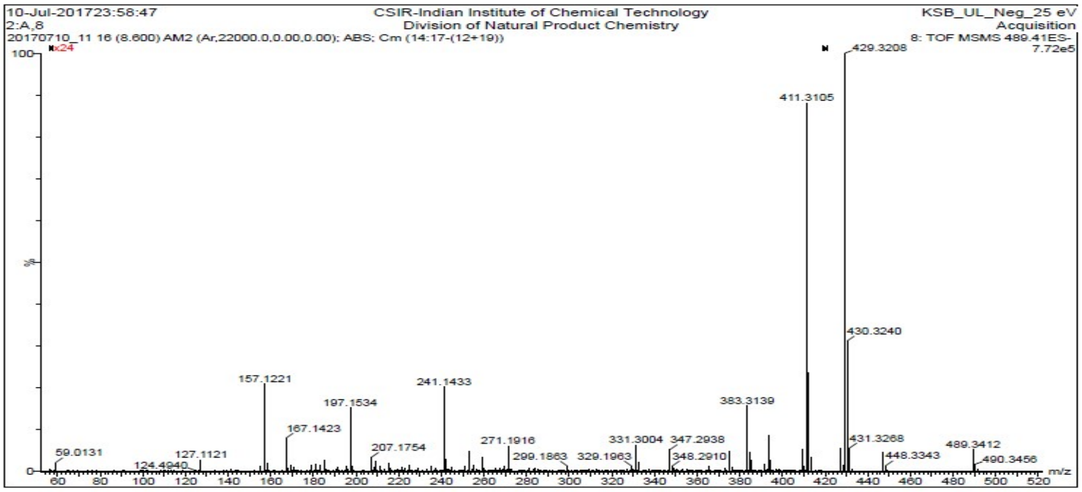

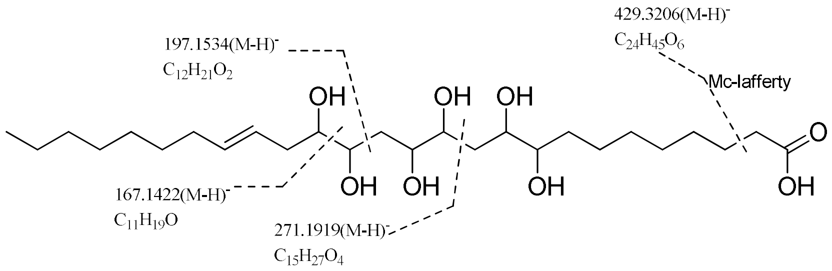

Figure S12. MS/MS spectrum and proposed fragmentation of compound **8**

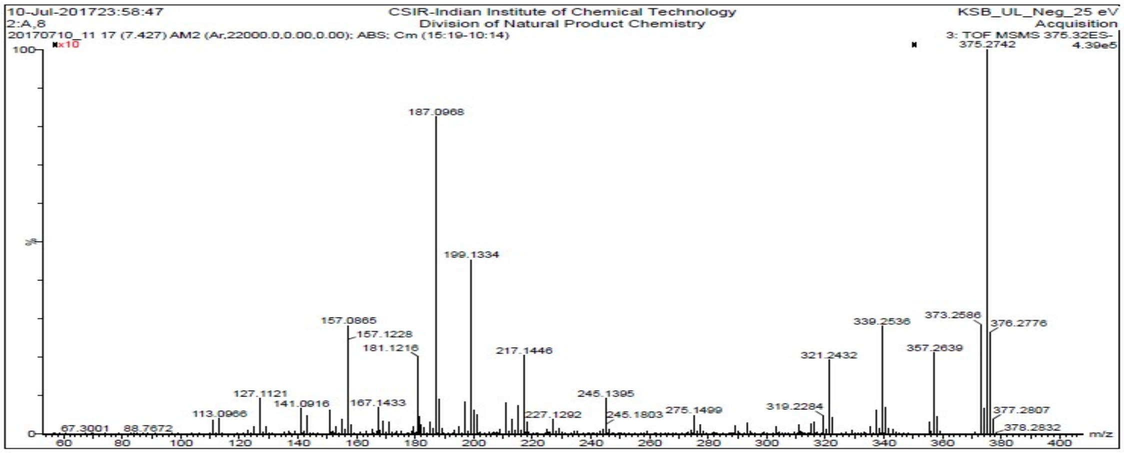

Figure S13. MS/MS spectrum of compound **9**

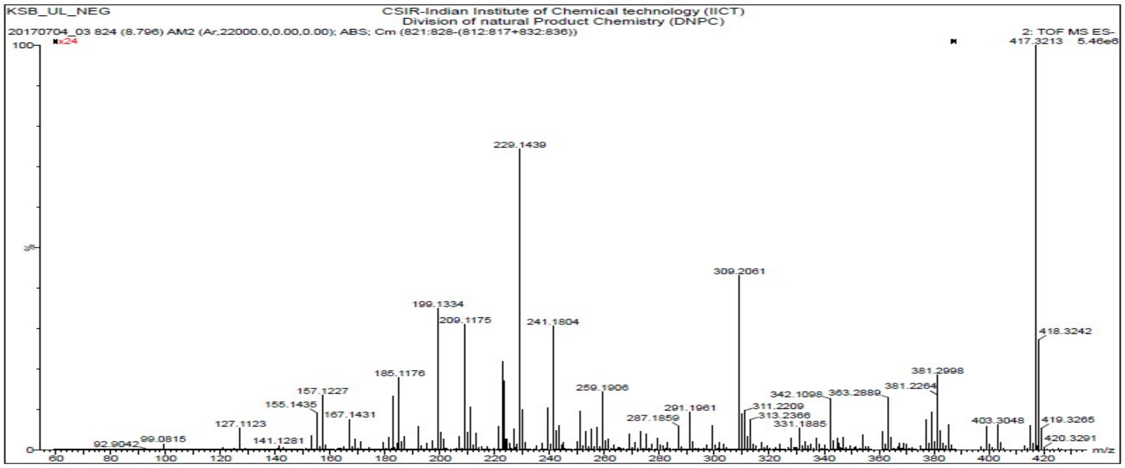

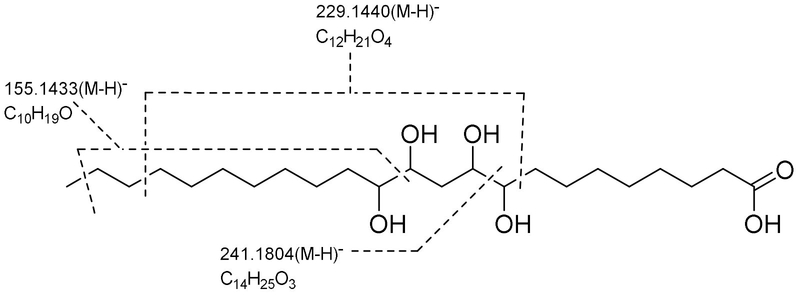

Figure S14. MS/MS spectrum and proposed fragmentation of compound **10**

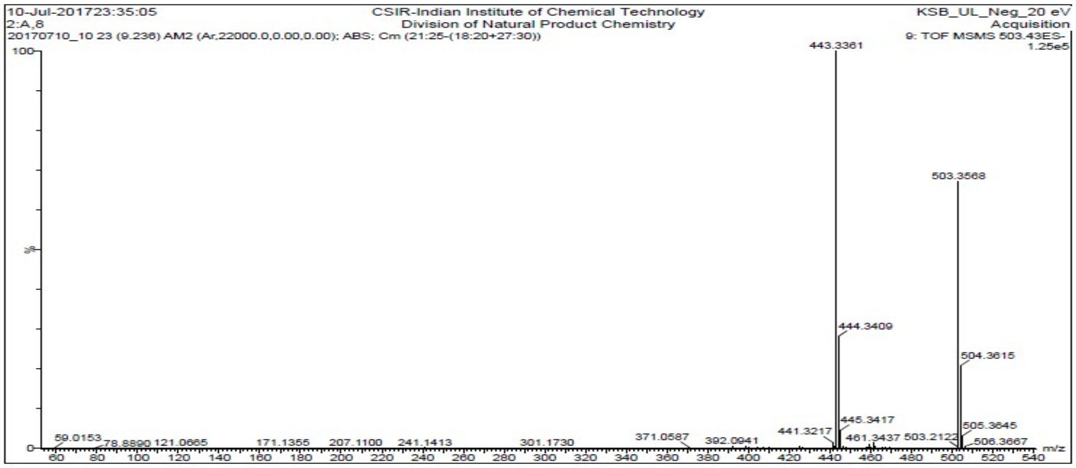

Figure S15. MS/MS spectrum of compound **11**

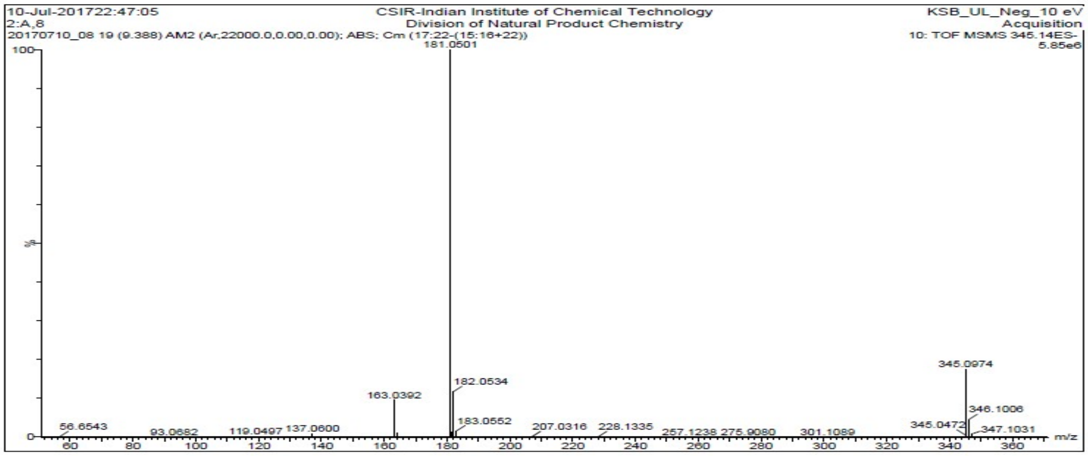

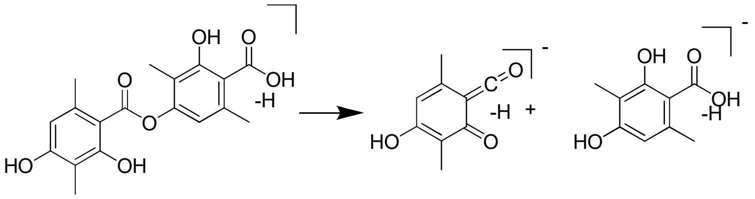

Figure S16. MS/MS spectrum and proposed fragmentation of compound **12**

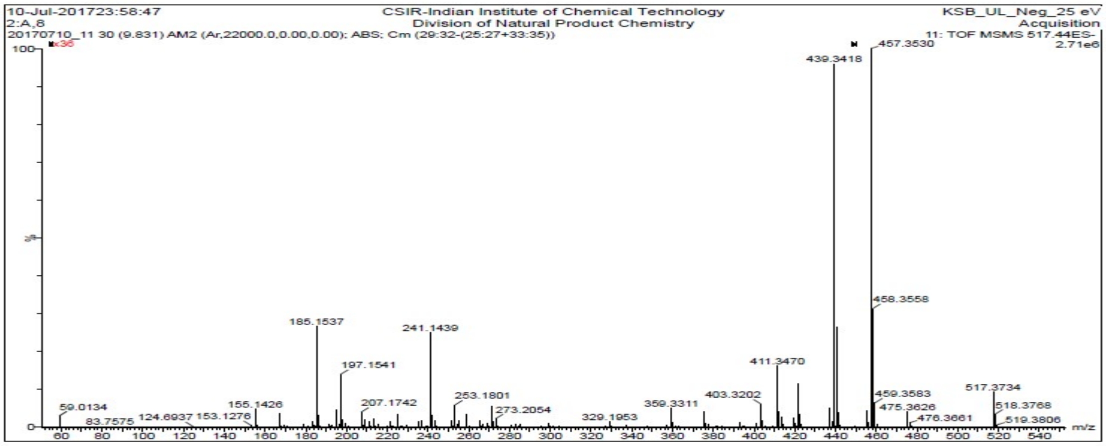

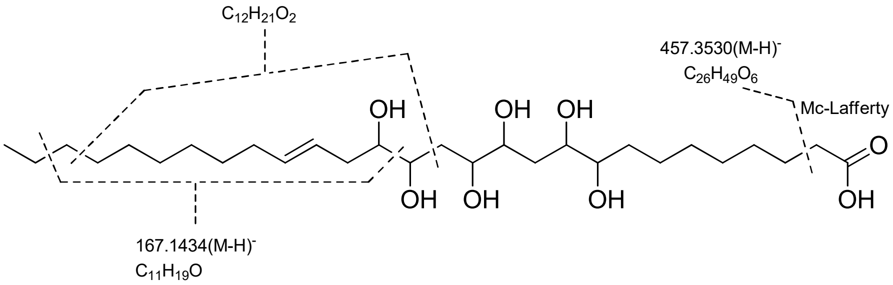

Figure S17. MS/MS spectrum and proposed fragmentation of compound **13**

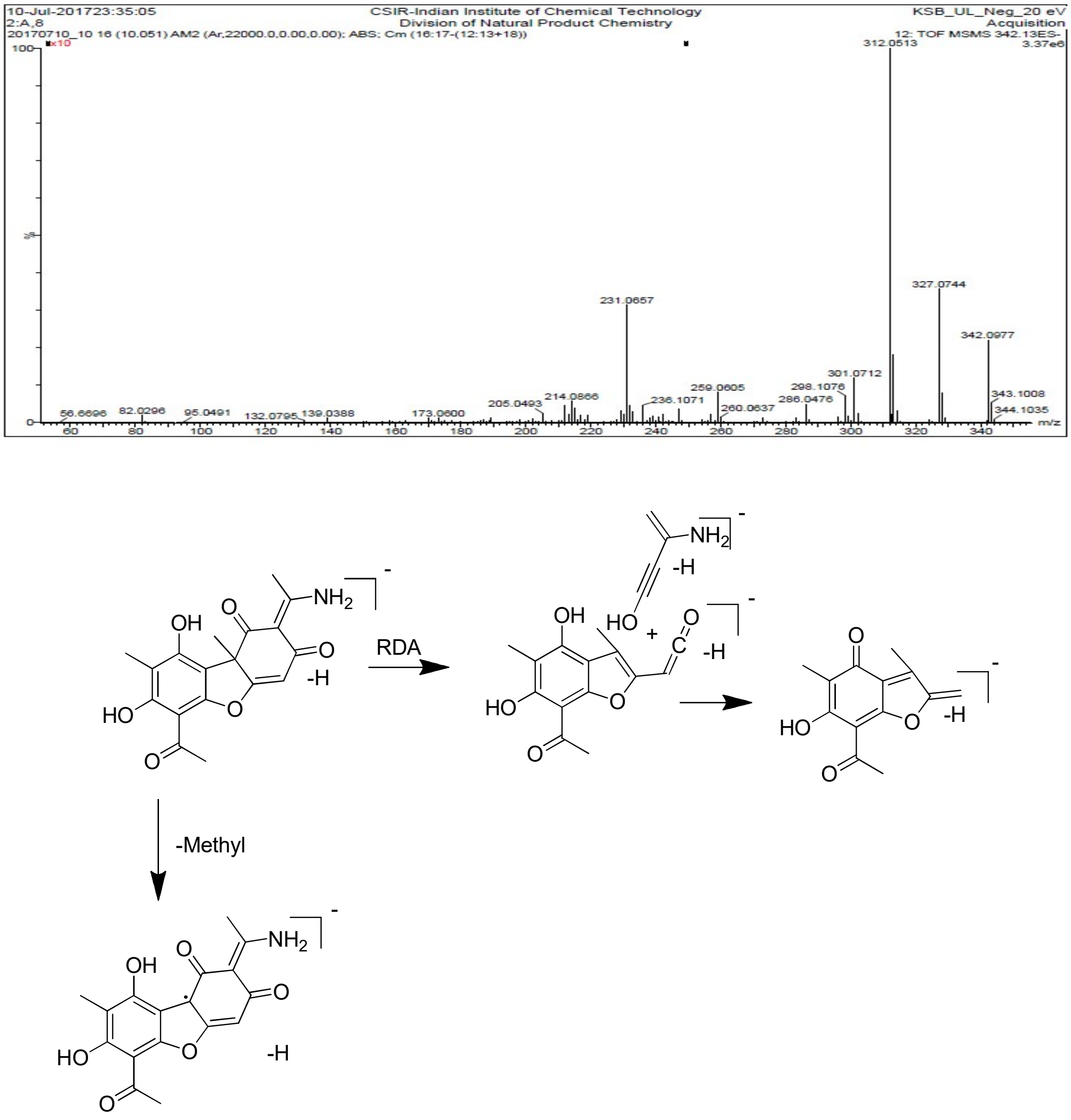

Figure S18. MS/MS spectrum and proposed fragmentation of compound **14**

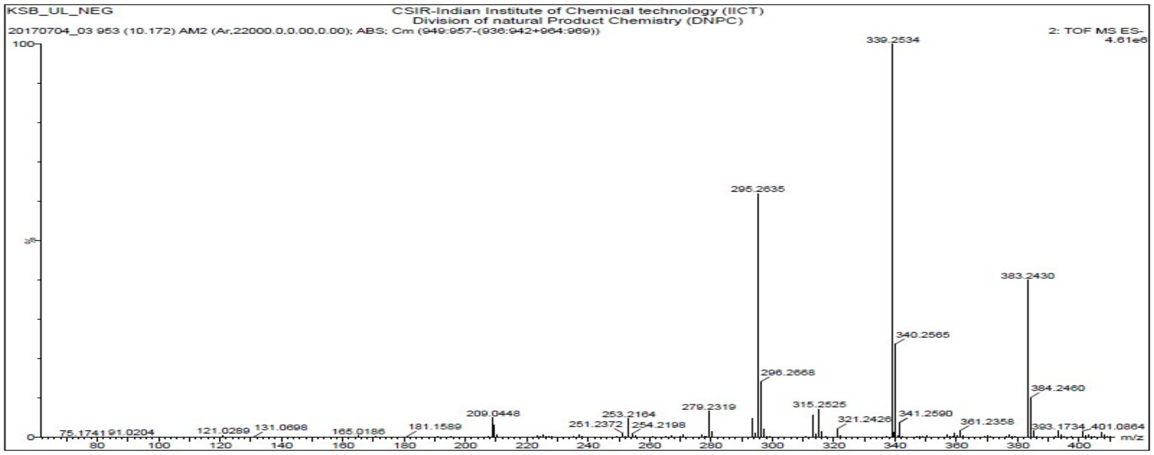

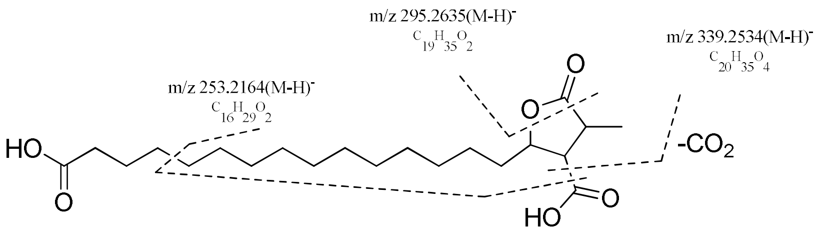

Figure S19. MS/MS spectrum and proposed fragmentation of compound **15**

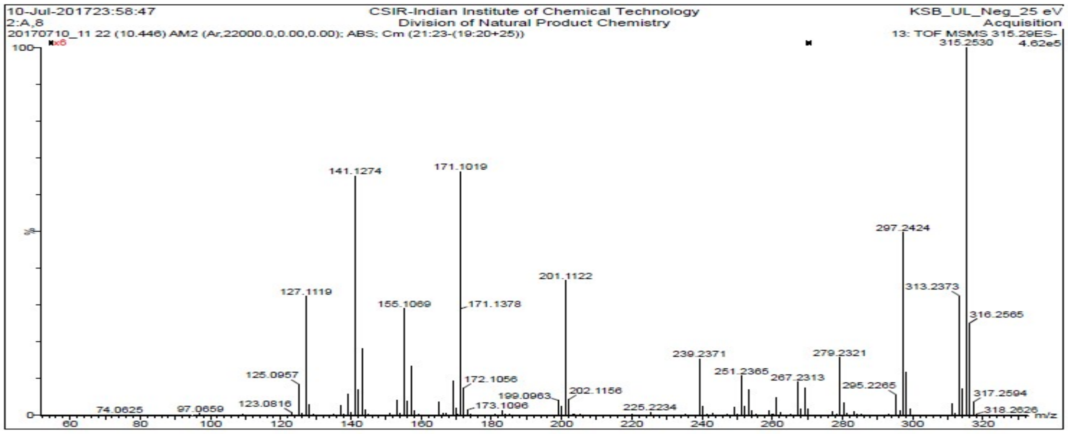

Figure S20. MS/MS spectrum of compound **16**

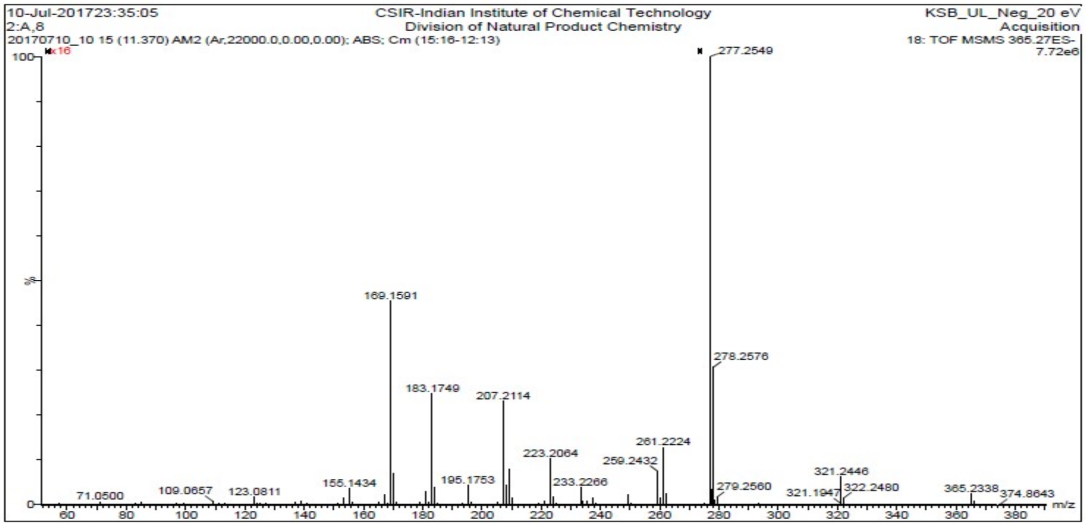

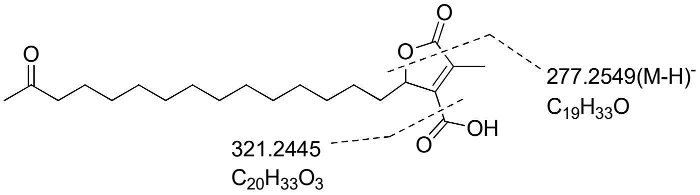

Figure S21. MS/MS spectrum and proposed fragmentation of compound **17**

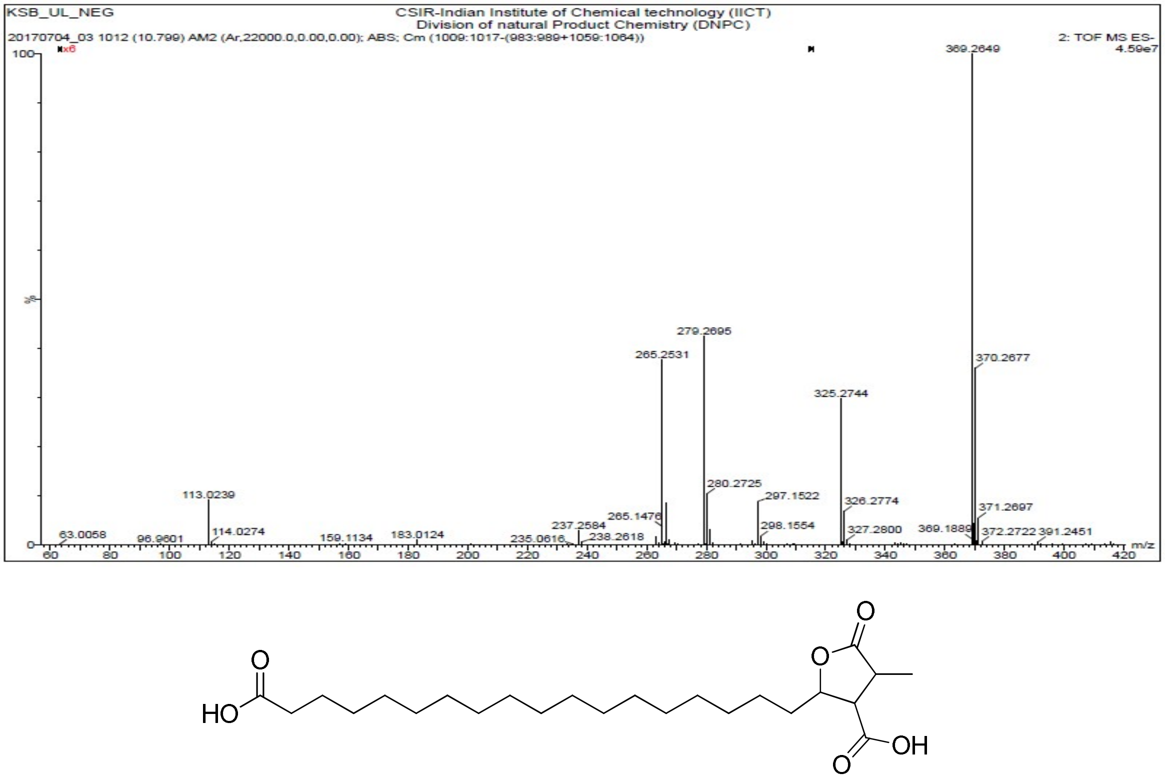

Figure S22. MS/MS spectrum and proposed fragmentation of compound **18**

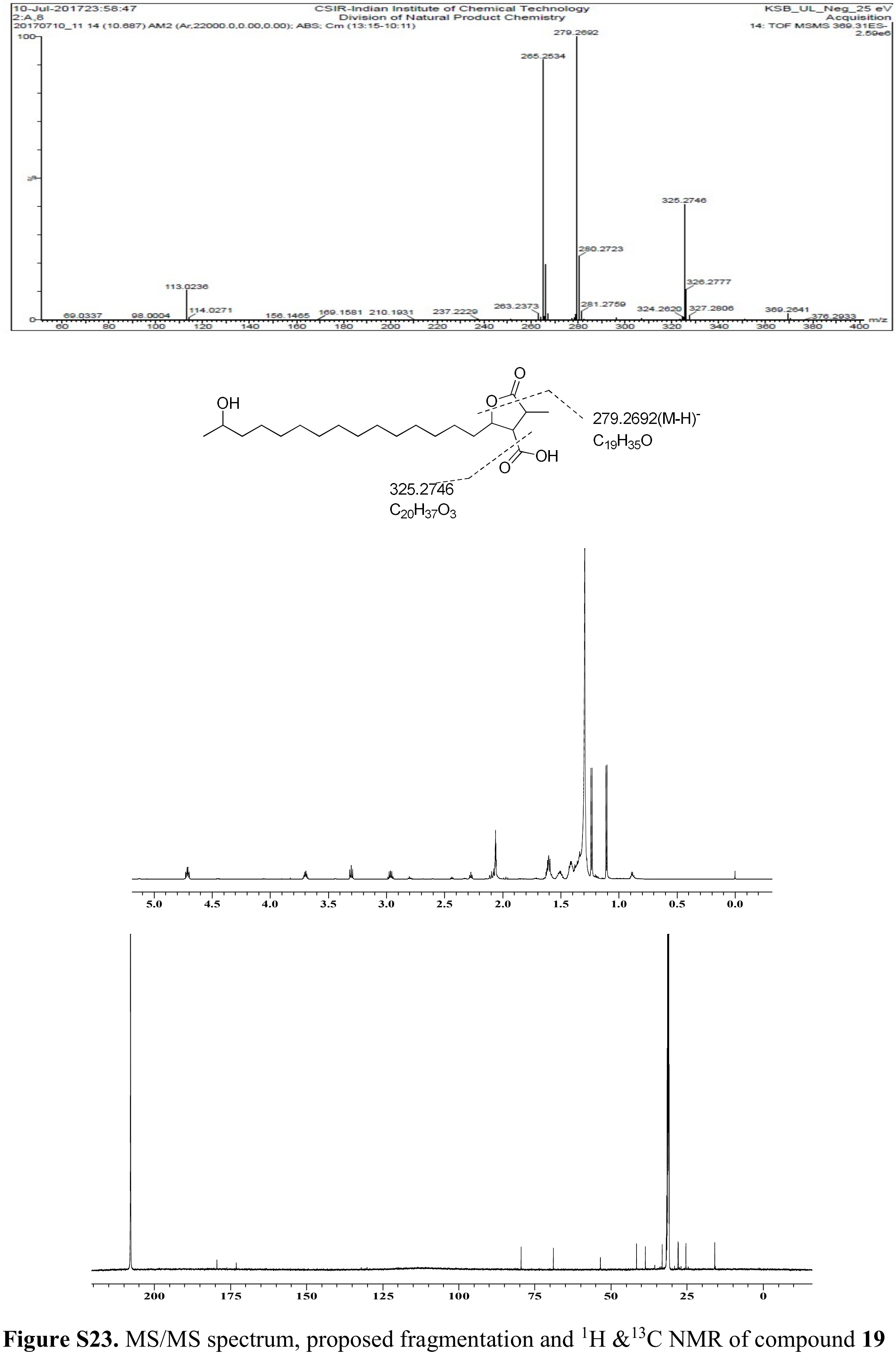

Figure S23. MS/MS spectrum, proposed fragmentation and ^{1}H &^{13}C NMR of compound **19**

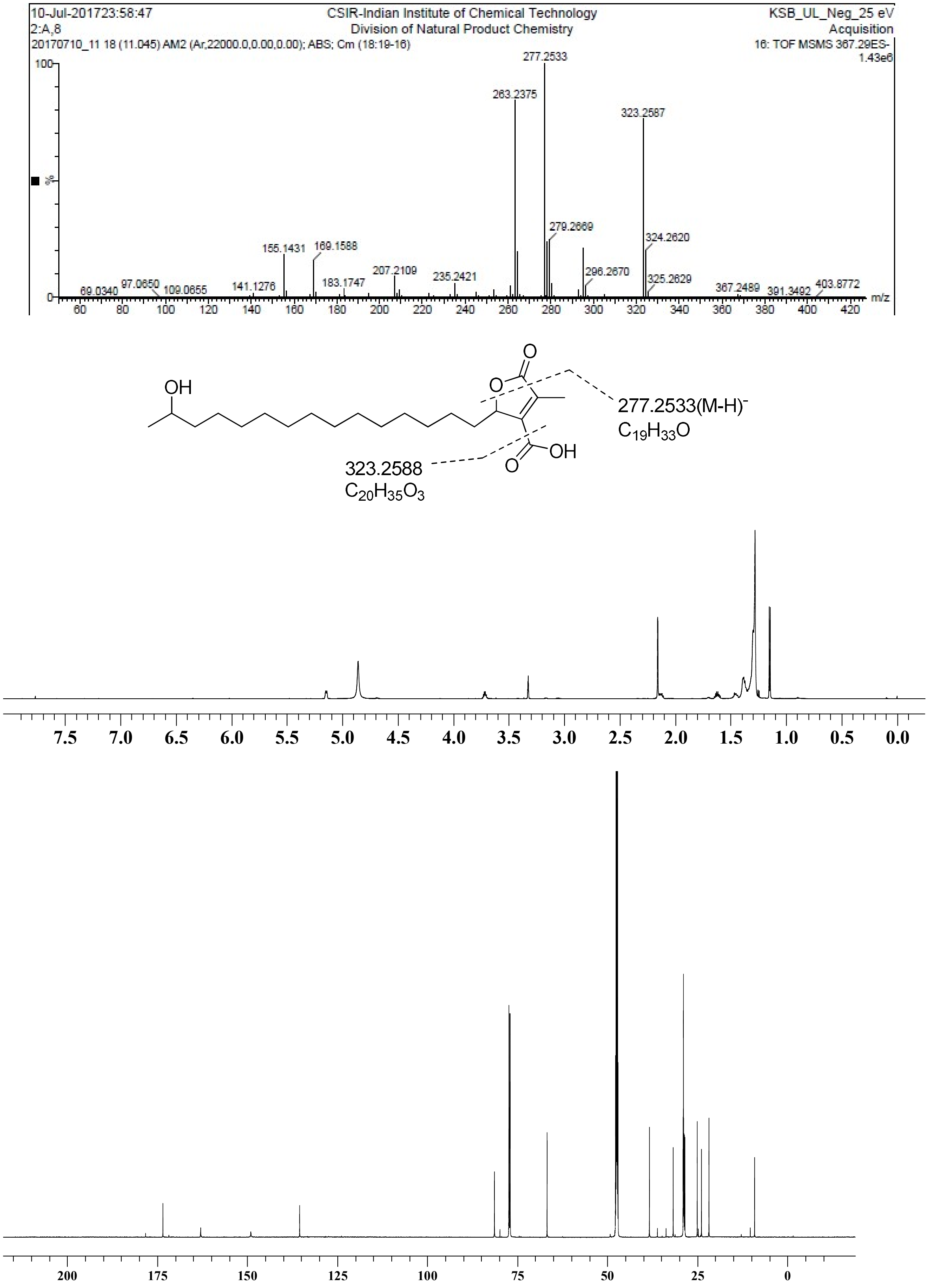

Figure S24. MS/MS spectrum, proposed fragmentation and ^{1}H &^{13}C NMR of compound **20**

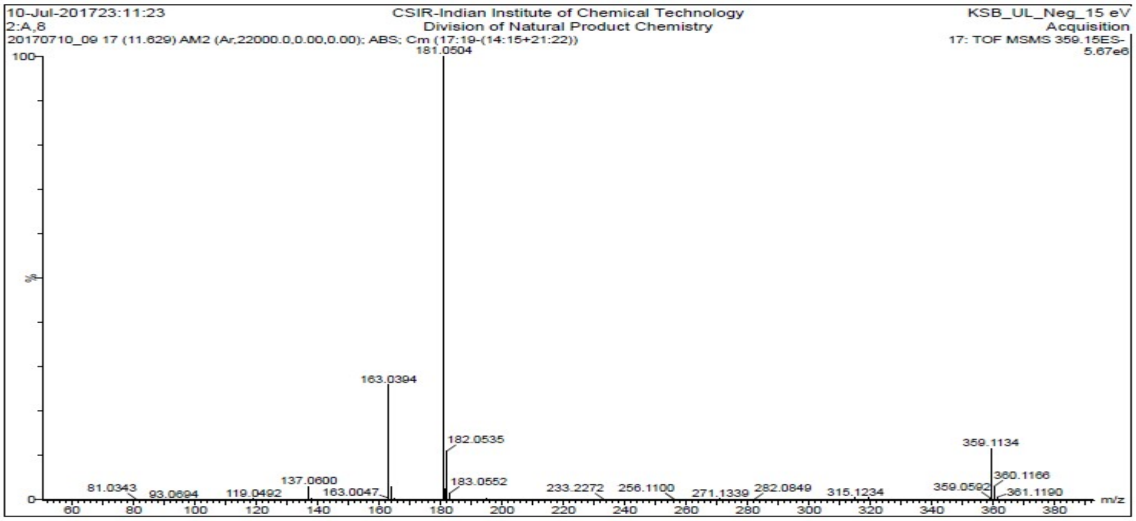

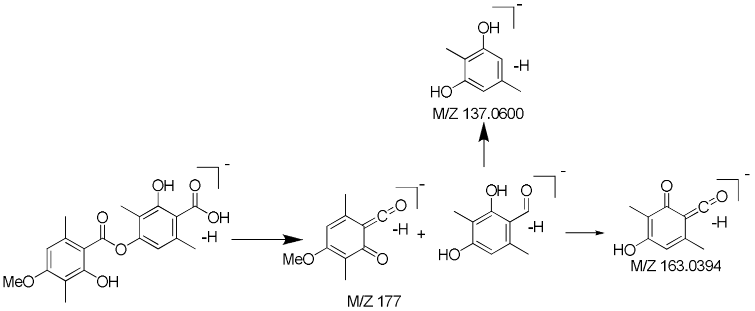

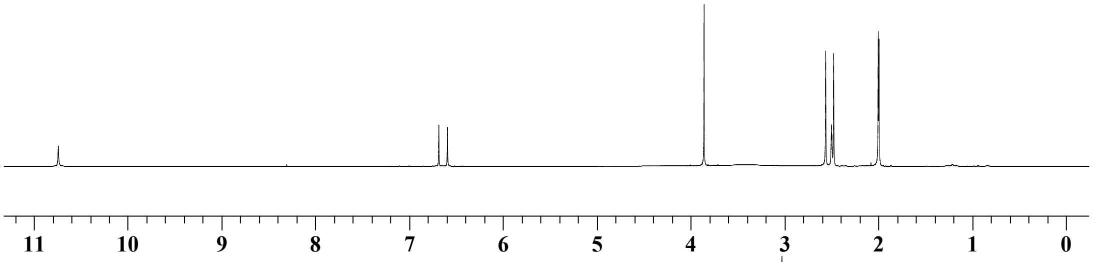

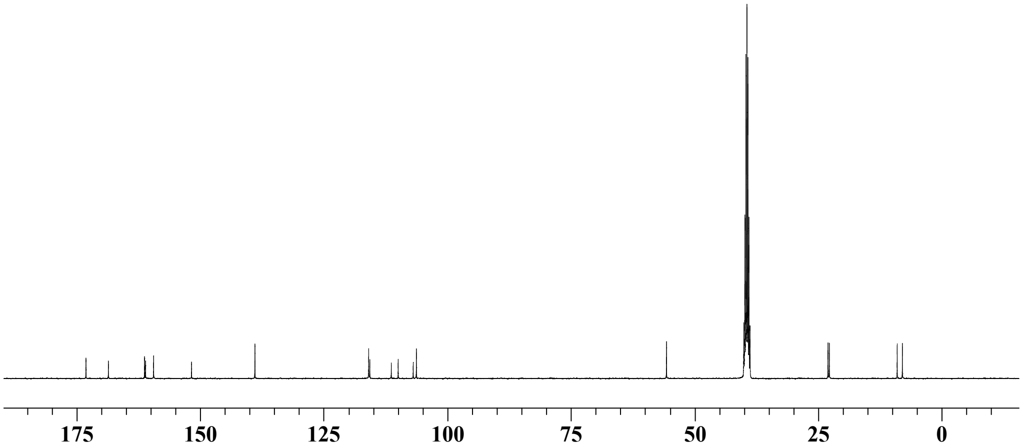

Figure S25. MS/MS spectrum, proposed fragmentation and ^{1}H &^{13}C NMR of compound **21**

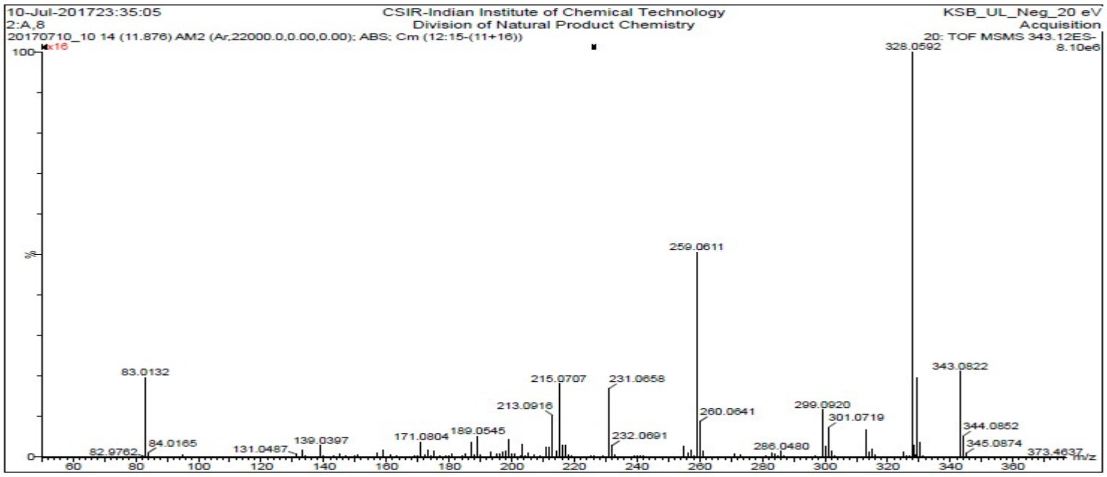

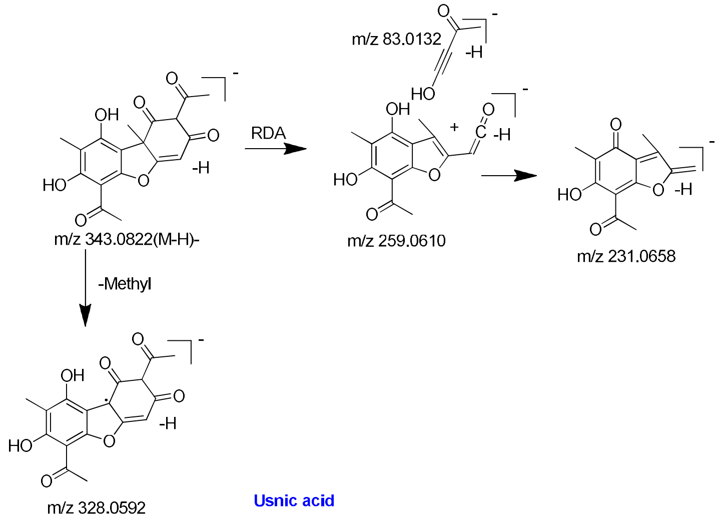

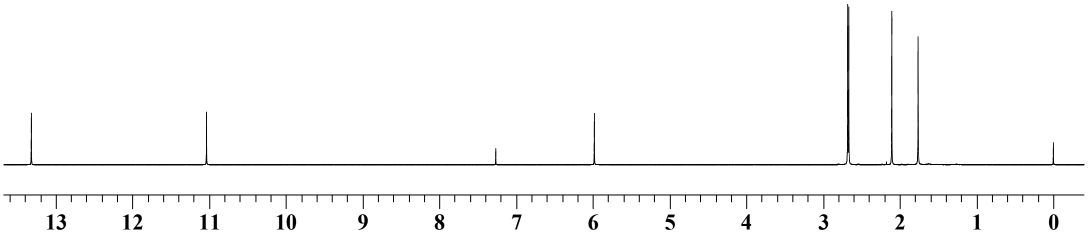

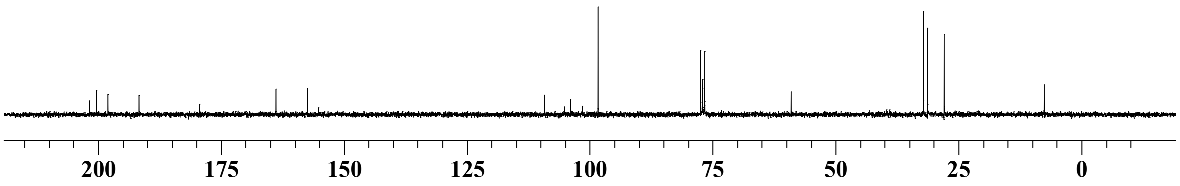

Figure S26. MS/MS spectrum, proposed fragmentation and ^{1}H & ^{13}C NMR of compound **22**

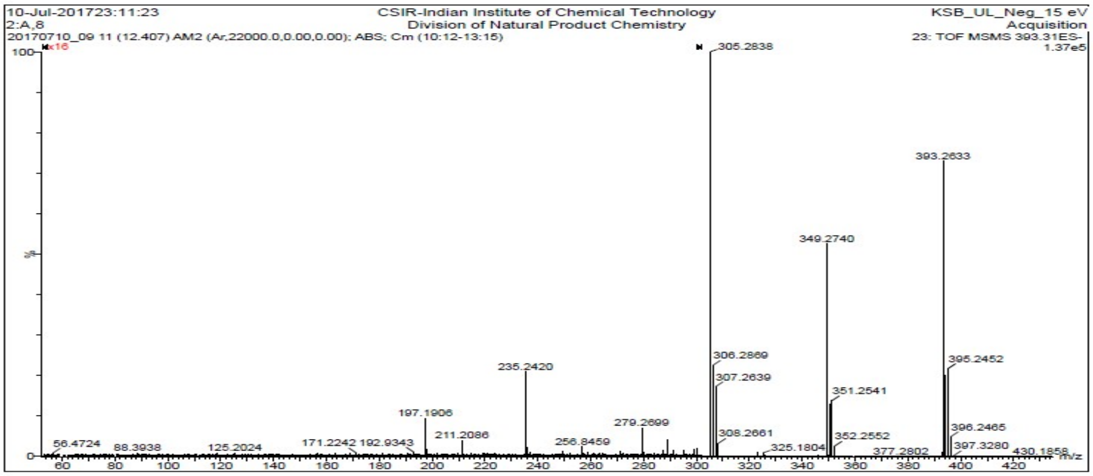

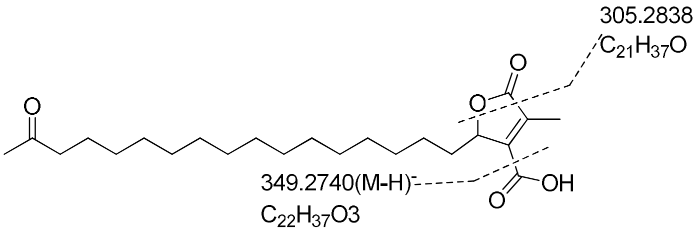

Figure S27. MS/MS spectrum and proposed fragmentation of compound **23**

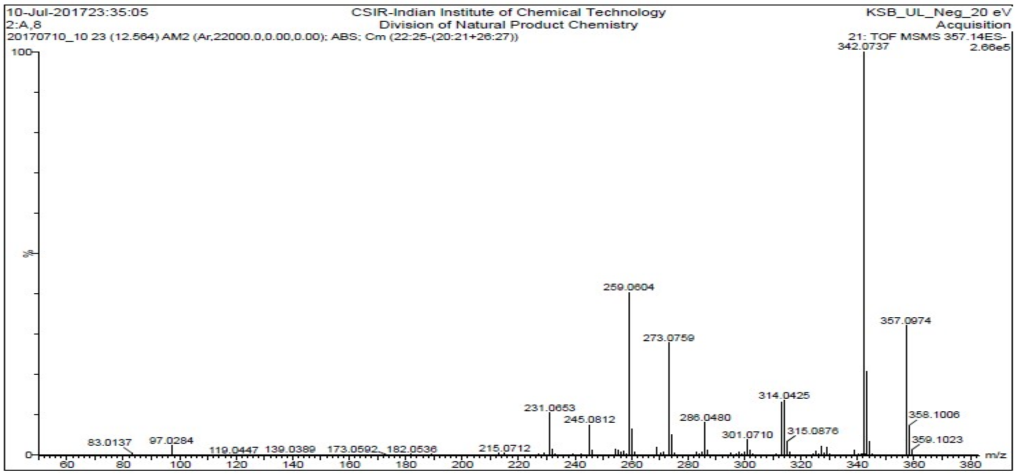

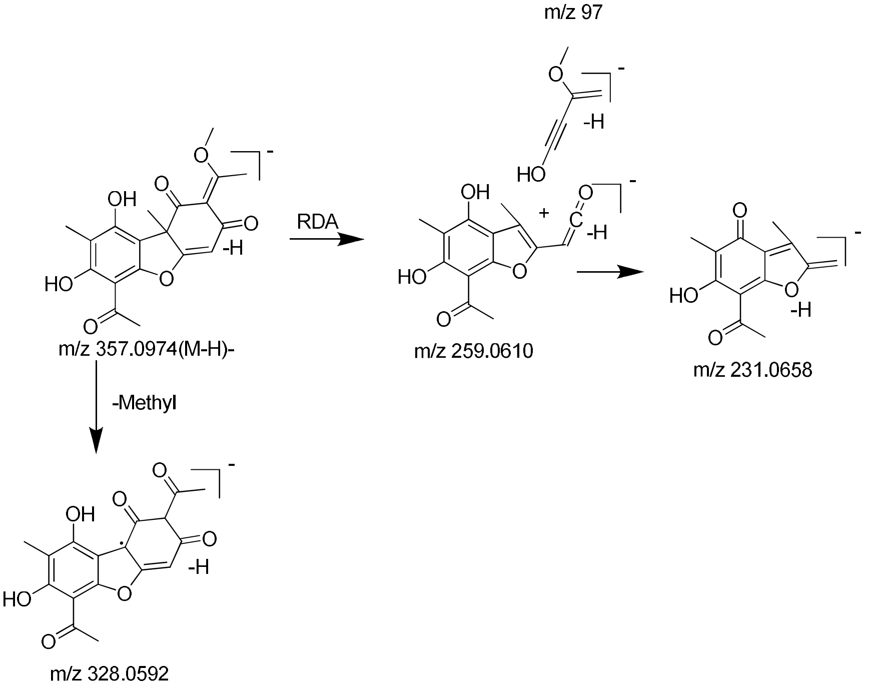

Figure S28. MS/MS spectrum and proposed fragmentation of compound **24**

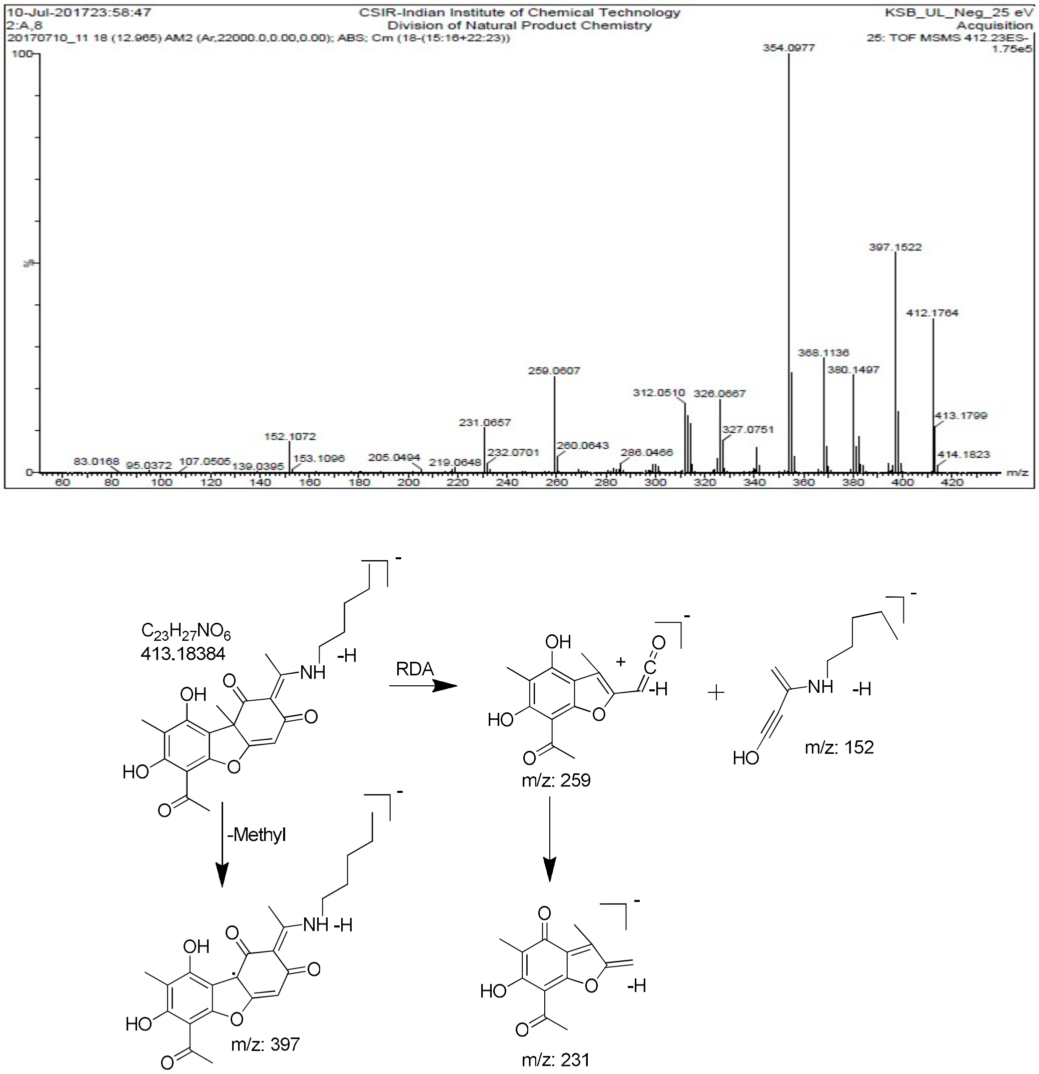

Figure S29. MS/MS spectrum and proposed fragmentation of compound **25**

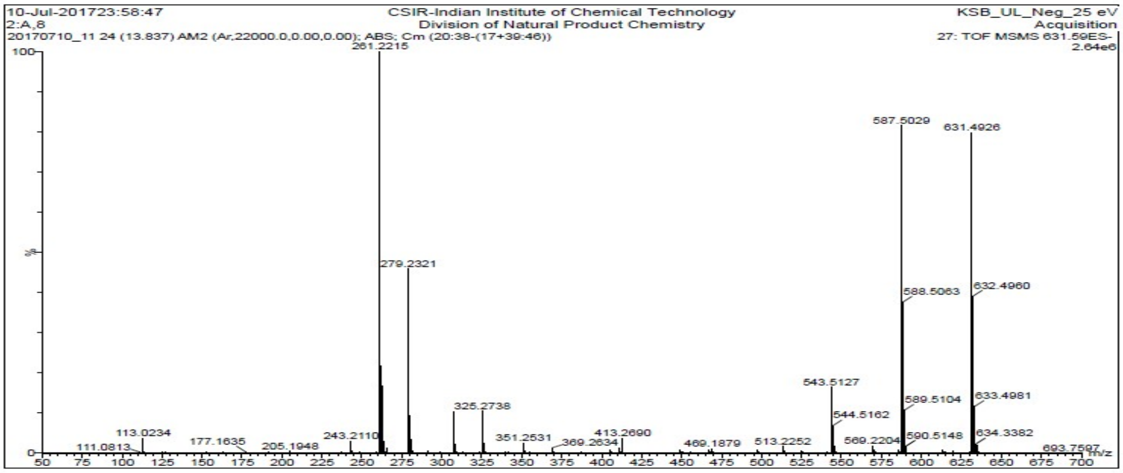

Figure S30. MS/MS spectrum of compound **26**
